# Common molecular links and therapeutic insights between type 2 diabetes and kidney cancer

**DOI:** 10.1371/journal.pone.0330619

**Published:** 2025-08-20

**Authors:** Reaz Ahmmed, Mohammad Amirul Islam, Md. Taohid Hasan, Arnob Sarker, Md. Ahad Ali, Md. Saiful Islam, Mst. Zafrin Sultana, Md. Nurul Haque Mollah

**Affiliations:** 1 Bioinformatics Lab (Dry), Department of Statistics, University of Rajshahi, Rajshahi, Bangladesh; 2 Department of Biochemistry and Molecular Biology, University of Rajshahi, Rajshahi, Bangladesh; Wenzhou Medical University, CHINA

## Abstract

**Introduction:**

Type 2 diabetes (T2D) is considered as a risk factor for kidney cancer (KC). However, so far, there is no study in the literature that has explored genetic factors through which T2D drive the development and progression of KC. Therefore, this study attempted to explore T2D- and KC-causing shared key genes (sKGs) for revealing shared pathogenesis and therapeutic drugs as their common treatments.

**Methods:**

The integrated bioinformatics and system biology approaches were utilized in this study. The statistical LIMMA approach was used based web-tool GEO2R to detect differentially expressed genes (DEGs) through transcriptomics analysis. Then upregulated and downregulated DEGs for T2D and KC were combined to obtained shared DEGs (sDEGs) between T2D and KC. The STRING database was used to construct the protein-protein interaction (PPI) network of sDEGs. Then Cytohubba plugin-in Cytoscape were used in the PPI network to disclose the sKGs based on different topological measures. The RegNetwork database was used in NetworkAnalyst to analyze co-regulatory networks of sKGs with transcription factors (TFs) and micro-RNAs to identify key TFs and miRNAs as the transcriptional and post-transcriptional regulators of sKGs, respectively. AutoDock Vina is a tool used for molecular docking. ADME/T properties were 24 assessed using pkCSM and SwissADME.

**Results:**

At first, 74 shared DEGs (sDEGs) were identified that can distinguish both KC and T2D patients from control samples. Through protein-protein interaction (PPI) network analysis, top-ranked 6 sDEGs (CD74, TFRC, CREB1, MCL1, SCARB1 and JUN) were detected as the sKGs that drive both KC and T2D development and progression. The most common sKG ‘CD74’ is associated with key pathways, such as NF-κB signaling transduction, apoptotic processes, B cell proliferation. Differential expression patterns of sKGs validated by independent datasets of NCBI database for T2D and TCGA and GTEx databases for KC. Furthermore, sKGs were found to be significant at several CpG sites in DNA methylation studies. Regulatory network analysis identified three TFs proteins (SMAD5, ATF1 and NR2F1) and two miRNAs (hsa-mir-1-3p and hsa-mir-34a-5p) as the regulators of sKGs. The enrichment analysis of sKGs with KEGG-pathways and Gene Ontology (GO) terms revealed some crucial shared pathogenetic mechanisms (sPM) between two diseases. Finally, sKGs-guided four potential therapeutic drug molecules (Imatinib, Pazopanib hydrochloride, Sorafenib and Glibenclamide) were recommended as the common therapies for KC with T2D.

**Conclusion:**

The results of this study may be useful resources for the diagnosis and therapy of KC with the co-existence of T2D.

## 1. Introduction

**Fig 1:**
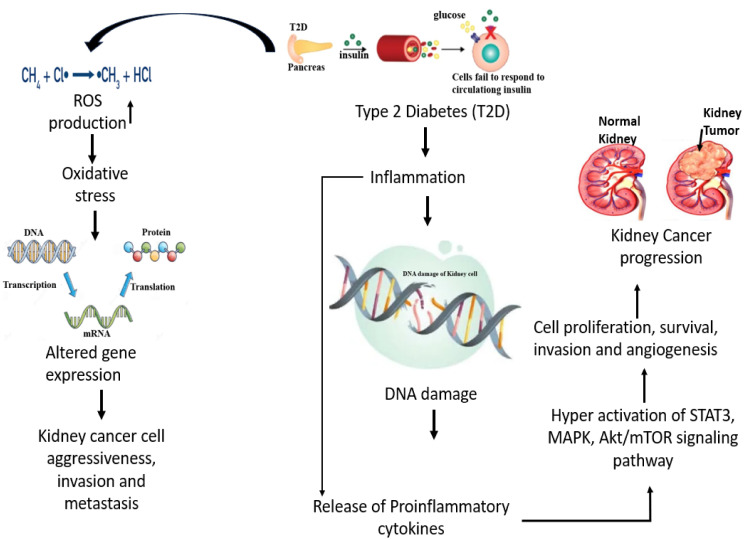
The Schematic diagram about the relationship between T2D and KC. It highlights how hyperglycemia and IR in T2D contribute to oxidative stress, inflammation, and altered immune responses, which may promote KC development.

Kidney cancer (KC) ranks among the most prevalent urological cancers [1]. It is a disease caused by uncontrolled proliferation of cells and spread throughout the body [2]. The renal parenchyma is the primary site where KC arises [3]. Among all cancers, KC ranks 14^th^ in the world and It is the 7^th^ most frequently occurring cancer in men and the 9^th^ in women [4–6]. In adults, renal cell carcinoma (RCC) is the 2^nd^ prevalent form of KC [7]. According to histopathology, the most prevalent subtypes of renal cell carcinoma are chromophobe (5%–10%), papillary (10%–15%), and clear cell (75%–85%) [4]. Despite significant diagnostic and treatment advancements, the 5-year survival rate for KC has only reached 10% [8]. Early-stage KC is treatable, but advanced or metastatic KC typically results in death [9]. Approximately 42% of KC patients died in 2020 [10]. Due to non-specific symptoms and absence of screening guidelines, most KC patients are diagnosed at an advanced-stage, prohibiting the possibility of surgical intervention [11]. One of the main causes of the rise in cancer-related mortality is older people [12,13]. In adults, 2–3% of malignant disorders are kidneys tumors [14]. Studies on KC have shown that it is generally a metabolic disease [5]. Conversely, T2D is a metabolic disorder as well, marked by dysregulation of genes, glucose, and lipid metabolism [15]. T2D is characterized by higher blood-glucose levels caused due to either insufficient insulin production by the pancreas or insulin resistance in the body [16,17]. The reasons underlying the elevated cancer risk in diabetic people may include increased oxidative stress, proinflammatory state, insulin resistance, and hyperinsulinemia [18]. The two most well-established risk factors for renal cell carcinoma are obesity and hypertension, and both are associated with type 2 diabetes through metabolic syndrome and insulin resistance [19]. According to some studies, T2D have an increased risk of developing KC [16,18–22]. More than 90% of all instances of diabetes are T2D, which is more prevalent in older people and worsens with age [23]. It has been estimated that up to 40% of people with T2D also have KC [24,25]. The above data indicates that T2D increases the risk of developing KC to the patients. The relationship between T2D and KC is shown schematically in Fig 1.

Multiple studies have been conducted to investigate the genetic factors contributing to T2D and KC separately. It is crucial to identify shared genes/proteins has been associated with T2D and KC in order to develop appropriate treatment plans for patients dealing with both of these complex conditions simultaneously. Doctors typically prescribe drugs that are specific to the patient’s disease [26]. As a result, they have to prescribe multiple drugs to the patients who are suffering from multiple diseases. However, polypharmacy can lead to drug-drug interactions (DDIs), which may cause adverse effects or toxicity, potentially worsening the patient’s health [27–30]. In such situations, doctors should aim to prescribe a smaller number of common drugs that effectively represent the specific medications of those diseases, thereby reducing toxicity. However, no research has yet to propose a common medication for the treatment of KC patients who are also suffering from T2D. Therefore, specific objectives of this bioinformatics-based study are to explore (i) T2D and KC causing shared key-genes (sKGs) highlighting their common pathogenetic mechanisms, and (ii) sKGs-guided repurposable common drug molecules for the treatment of KC with T2D.

## 2. Materials and methods

### 2.1. Sources of data and their descriptions

As shown below, we employed both transcriptomic data and meta-data related to T2D and KC in this study:

#### 2.1.1. Collection of transcriptomic datasets to discover potential sKGs.

To determine which T2D stimulates KC, we take two types of datasets (control vs. KC and control vs. T2D). We took two KC datasets (GSE15641 [31], GSE38424 [32]) and two T2D datasets (GSE29226 [33], GSE25724 [34]) from Gene Expression Omnibus (GEO) database of the National Center for Biotechnology Information (NCBI). The KC dataset with accession ID GSE38424 consisted of 4 renal-cell carcinoma (RCC) and 4 control. The KC dataset with accession ID GSE15641 consisted of 32 clear-cell RCC (cRCC), 6 chromophobe RCC (chrRCC), 11 papillary RCC (pRCC), 12 Oncocytoma (OC), 8 transitional cell carcinoma (TCC) and 20 control, where ccRCC, pRCC, chrRCC, OC, and TCC were considered as case group.

**Table 1** presents comprehensive descriptions of the transcriptomics datasets utilized in the study.

**Table 1:** Data sources and descriptions.

Datasets	Country	Platform	Cases	Control
GSE38424 [32]	Singapore	GPL10558	4(KC)	4
GSE15641 [31]	USA	GPL96	69(KC)	23
GSE29226 [33]	India	GPL6947	12(T2D)	12
GSE25724 [34]	Italy	GPL96	6(T2D)	7

#### 2.1.2. Collection of drug molecules.

In order to identify possible medication for the treatment of patients with T2D and KC, we collected 156 drug molecules reported to be associated with both T2D and KC linked meta-drug molecules from the published articles and online database (Table S1 in S1 File).

### 2.2. Identification of Differentially Expressed Genes (DEGs) between case and control groups

We used the GEO2R online tool to find differential expression genes (DEGs) between cancer samples and normal samples using the linear models for microarray (LIMMA) [35] approach for each of four datasets. Each dataset was normalized using RMA software. We selected common upregulated DEGs between T2D and KC as well as common downregulated DEGs using the threshold at log_2_FC. > 1 and log_2_FC < −1, respectively. Then we combined both upregulated and downregulated DEGs to get final DEGs-set. We consider common sDEGs and then identified common key gene among T2D and KC. The average of log_2_ fold-change (aLog_2_FC) values and the adjusted *P*.value of the *k*^th^ differentially expressed gene (DEG*_k_*) are combined to define DEG_*k*_ as follows:


$${\text{ DEG}}_{k}=\left\{ \begin{array}{c} DEG(Up),\;if\;adj.p.value<0.05\;and\;a{Log}_{2\;}{FC}_{k}>+1.0\\ \;\\ DEG(Down),if\;adj.p.value<0.05\;and\;a{Log}_{2\;}{FC}_{k}<-1.0 \end{array}\right.\;$$

where aLog_2_FC_*k*_ [12,36] is computed as


$$aLog_{2\;}FC_{k}=\{\;\;\;\begin{array}{c} {}*20l\frac{1}{m_{1}}\sum_{i}^{m_{1}}log_{2}(z_{ki}^{D})-\frac{1}{m_{2}}\sum_{j}^{m_{2}}log_{2}(z_{kj}^{C}),\;\;\;\;\;\;if\;m_{1}\neq m_{2}\\ \frac{1}{m}\sum_{i}^{m_{1}}log_{2}\left(\frac{z_{ki}^{D}}{z_{kj}^{C}}\right),\;\;\;\;\;\;\;\;\;\;\;\;\;\;if\;\;m_{1}=m_{2}\;=m\;\;\;\;\;\;\;\;\;\;\;\;\;\;\;\;\;\;\; \end{array}\;\;\;\;\;\;\;\;\;\;\;\;\;\;\;$$ (1)

Here $z_{ki}^{D}$ and $z_{kj}^{C}$ represent the expression values of *k*th gene for the *i*th case and *j*th control samples, respectively. Thus, we computed common upregulated and downregulated DEGs for both T2D and KC.

### 2.3. Identification of T2D and KC causing shared key genes (sKGs)

Generally, a protein cannot work alone. In order to perform a function of a protein, it interacts with other one or more proteins within the cells. The protein-protein interaction (PPI) network is widely used in identifying diseases related key-genes (KGs) from a set of differentially expressed genes (DEGs) [37–40]. The PPI network is created by measuring the distance “D” between the proteins (m, n) as follows:


$$𝐃\text{(m,n)}=\;\frac{2\;|N_{m}\cap N_{n}|}{\left|N_{m}\right|+|N_{n}|}\mathrm{\backslash ]}$$

where Nm and Nn are the corresponding nearby sets of m and n. The distance metric “D” represents the degree of functional or topological separation between two proteins within the interaction network. A lower “D” value indicates a closer functional relationship or stronger connectivity, suggesting that the proteins may share common biological pathways or regulatory mechanisms. Conversely, a higher “D” value implies weaker connectivity or distinct functional roles, potentially indicating disparate biological functions or minimal direct interaction [41]. In order to construct PPI network for DEGs, STRING [42] is popular database [43–46]. In this study, a high-confidence interaction score cutoff of 0.700 was applied to construct PPI network. The node of a PPI network shows a protein, and the edge shows how proteins interact with one another. In order to select KGs from the PPI network by prioritizing different topological measures, the CytoHubba [47] plugin in Cytoscape [48] is a popular web-tool [13,14]. This study also considered this web-tool and database to select the shared KGs (sKGs) from sDEGs by prioritizing different topological measures with threshold of Betweenness = 318.665, BottleNeck = 6, Closeness = 38.56, Radiality = 4.21, MNC = 18, EPC = 32.413, and Stress = 530.

### 2.4. Verification on the association of sKGs with T2D and KC by using independent datasets and databases

Using the independent datasets from the NCBI, GTEx and TCGA databases, box plot analysis was used to validate the differential expression patterns of sKGs in both disease (KC and T2D). We verified the difference sKG expression patterns between KC and control samples using the GTEx and TCGA databases in the UALCAN web-tool [49]. We used two separate datasets with accession IDs GSE16449 [50] and GSE20966 [51] from the NCBI GEO database to verify the differing sKG expression patterns between T2D and control samples.

### 2.5. Regulatory analysis of sKGs

We used NetworkAnalyst [52,53] platform-based RegNetwork [54]databases and performed the study of the co-regulatory network between sKGs, TFs, and miRNAs in order to identify key micro-RNAs (miRNAs) and transcription factors (TFs) of sKGs. It was determined that the Cytoscape software [55] represented the networks more accurately. Seven independent topological analyses (Betweenness [56], BottleNeck [57], Closeness [58] MNC [59], Radiality [60], EPC [61] and Stress [62]) were used to choose the core regulators.

### 2.6. Revealing common pathogenetic processes of T2D and KC with sDEGs

To investigate biological processes (BP), cellular components (CC), molecular functions (MF), and pathways of sKGs, sKGs-set enrichment studies were conducted using gene ontology (GO) keywords and Kyoto encyclopedia of genes and genomes (KEGG) pathways [63–66]. To find KEGG-pathways or highly enriched GO-functions (BPs, CCs, MFs,) by the sKGs-set, a 2x2 contingency table was created in Table 2.

**Table 2:** A 2 × 2 contingency table for sKGs-set enrichment analysis.

Status of Annotation	sKGs (proposed)	Not-sKGs	Marginal total
Annotated gene-set ($A_{j}$) in *i*th group	*m*_*j*_	*N*_*j*_ *– m*_*j*_	*N*_*j*_
Not annotated ($A_{j}^{c})$ in *i*th group	*q – m*_*j*_	*Q – N*_*j*_ *– q + m*_*j*_	*Q – N*_*j*_
Marginal total	*q*	*Q – q*	*Q* (Total grand)

where *N*_*j*_: total number of annotated genes in *j*th group ($A_{j};\;$*j* = 1, 2,…,*r*); *Q*: total number of annotated genes in all groups $A={⋃}_{j=1}^{r}A_{j}=A_{j}{\mathrm{\backslash bigcupA}}_{j}^{c}$ such that $Q\leq \sum_{j=1}^{r}N_{j}.$, since some genes are annotated in different groups. Here *q:* total number of sKGs, *m*_*j*_: number of sKGs belonging to *Aj*. The Database for Annotation, Visualization, and Integrated Discovery (DAVID) [67] was used to calculation of the *p*-value using the Fisher exact test based on hypergeometric distribution [68], in order to identify the highly enriched GO terms or KEGG pathways associated with the sKGs.

### 2.7. Methylation analysis of sKGs in KC

MethSurv [69] and UALCAN [70] was employed to investigate the methylation status of the sKGs in KC. β-values, which varied from 0 to 1, were used to express the degree of DNA methylation. The values are found using the formula N/ (N + O + 100). Here, N and O represent completely methylated and completely unmethylated intensities, respectively. In our analysis, we utilized normalized β-values obtained from both MethSurv and UALCAN databases.

### 2.8. Homology modeling

It was used to predict the three-dimensional (3D) structure of some receptor proteins that are unavailable in Protein Data Bank (PDB) [71]. A protein sequence in FASTA format or its reference number from UniProt database [72] is submitted to SWISS-MODEL for predicting the 3D structure of that protein. Qualitative Model Energy Analysis (QMEAN) [73] and QMEANDisco analysis [74] were used to assess the quality of the protein structure. The Ramachandran plot of the receptor protein was created using ProCheck [75] to confirm the expected 3D structures for molecular docking analysis.

### 2.9. Molecular docking

We looked at molecular docking studies of target receptors and meta-drug-agents to find FDA-approved medications that could be used to treat KC and T2D. As possible drug target receptors, the sKGs-mediated proteins and their regulatory TFs proteins were chosen in this example. Meta-drug agents were extracted from the published literature. The three-dimensional structures of every receptor protein were extracted using the Protein Data Bank (PDB) [76] and SWISS-MODEL [77] databases. From the PubChem database, the 3D structures of all 156 meta-drug compounds were selected [78]. Drug energy minimization is performed by Avogadro software (https://avogadro.cc/). This step helps predict the preferred structure of a molecule and its interactions with target sites [79]. MMFF94 is used as the force field. MMFF94 helps generate different conformations of a molecule by rotating around bonds and adjusting bond angles and lengths [80]. Steepest descent algorithm used in energy minimization. Using Swiss PDB view [81] and AutoDock Vina [82,83], protein receptors were created by including charge added and reducing energy, respectively. After selecting the appropriate chemicals to interact with the target proteins, molecular visualization is essential in model analysis. BIOVIA Discovery studio 2021 was used to view and analyze the docking outputs [84]. Let B_uv_ represent the binding affinity between the vth drug agent (v = 1, 2,..., s) and the uth receptor protein (u = 1, 2, …, r). To choose the top some drug molecules as possible candidate-drugs, the receptor proteins were sorted by decreasing-order of row average $\frac{1}{r}\sum_{v=1}^{r}B_{uv,}$ *j* = 1,2,…,r, and drug molecules were arranged by decreasing order of column average $\frac{1}{s}\sum_{u=1}^{s}B_{uv},$ *v* = 1,2,…,s.

### 2.10. ADME/T analysis

We used absorption, distribution, metabolism, excretion, and toxicity (ADME/T) study to look into the pharmacokinetics characteristics of the chosen drug compounds. The Lipinski rules fulfillment of drug likeness properties (such as: number of hydrogen bonds donor and acceptor, rotatable bond, molecular weight, LogP value, etc.) is assessed using the SCFBio web tool [85]. The ADMET properties were calculated using the pkCSM [86] and SwissADME [87] online databases. The optimum structures of medicinal substances were used in SMILES formats for the ADME/T computations.

### 2.11. The workflow from transcriptomics analysis to drug discovery

Unraveling Shared Molecular Mechanism between T2D and KC, and Therapeutic Indication: Insights from Bioinformatics and System Biology Analysis workflow is displayed in Supplementary Figure S1 in S1 File.

## 3. Results

### 3.1. DEGs identification

In each gene-expression dataset, we identified DEGs between case (T2D and KC) and control (normal) samples using the LIMMA with an r-package with an adjusted p value <0.05 and |aLog2(FC)| > 1 as described in section **2.2**. In the instance of the KC dataset, we identified 1421 and 3589 downregulated DEGs, and 4475 and 2983 upregulated DEGs, respectively, from the GEO datasets with the IDs GSE15641, and GSE38424. Common DEGs (cDEGs) of KC were found to be 559 upregulated and 130 downregulated (Table S2 in S1 File). Using the GEO datasets GSE25724 and GSE29226, we identified 2,651 and 424 upregulated DEGs, respectively, along with 3,032 and 1,763 downregulated genes in the T2D datasets. We identified 225 downregulated and 33 upregulated cDEGs associated with T2D (Table S3 in S1 File).

### 3.2. Shared DEGs (sDEGs) identification

Based on two transcriptomics datasets, we discovered 559 upregulated and 130 downregulated DEGs for KC in the previous section. Similarly, using two transcriptomics datasets, 33 upregulated and 225 downregulated DEGs were found for T2D. As a result, we take consideration of 74 sDEGs overall for T2D and KC (Table S4 in S1 File).

### 3.3. sKGs identification from sDEGs

**Fig 2:**
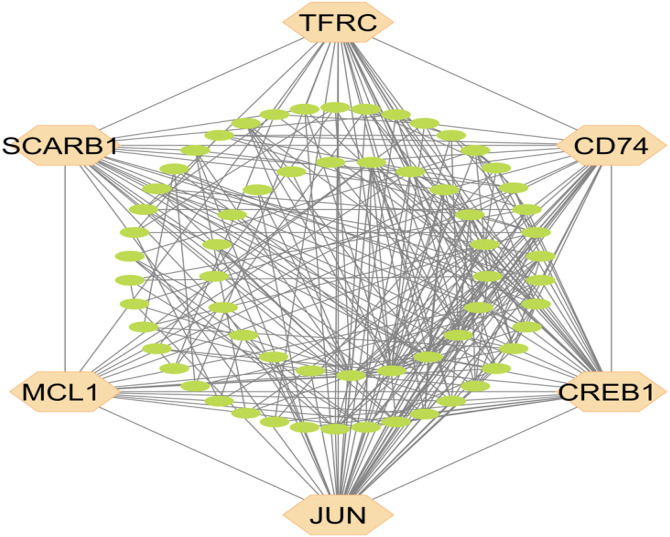
The Protein–protein interaction (PPI) network of shared DEGs (sDEGs) for T2D on KC to identify sKGs. Orange colour nodes indicate the sKGs. This network highlights the complex interactions among sDEGs and identifies sKGs. Orange colour nodes indicate the sKGs. These sKGs may play critical roles in the molecular crosstalk linking T2D and KC pathogenesis.

The PPI network, comprising sDEGs with 74 nodes and 295 edges (Fig 2). Utilizing 7 topological measures (BottleNeck, Closeness, Betweenness, Radiality, MNC, EPC, and Stress) within the PPI networking, we identified the top six sKGs: JUN, CD74, TFRC, CREB1, MCL1, and SCARB1 (Table S5 in S1 File).

### 3.4. Verification of sKGs with T2D and KC by using independent datasets and databases

Based on the expression patterns in the GTEx and TCGA databases, we analysed the expression pattern using a box plot analysis that indicate five sKGs (TFRC, MCL1, SCARB1, CD74 and JUN) are upregulated and sKGs (CREB1) are downregulated (Supplementary Figure S2A in S1 File) in KC. We discovered with independent dataset Supplementary Figures S2B in S1 File shows that five sKGs (TFRC, MCL1, SCARB1, CD74 and JUN) are upregulated in T2D, while the other sKGs (CREB1) are downregulated, supporting our results.

### 3.5. Analysis of the regulatory network associated with sKGs

**Fig 3:**
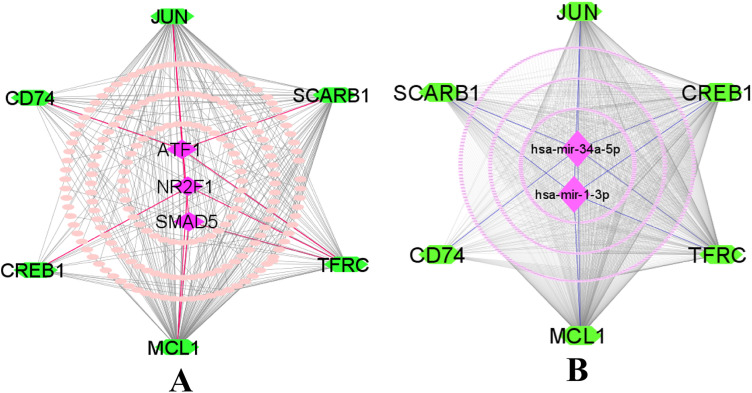
sKGs regulatory network (A) The JASPAR database-based on sKGs-TFs interaction network. (B) The TarBase database-based on miRNA-sKGs interaction network. sKGs are shown as green color octagons in both A and B, while TFs and miRNAs are displayed as pink color hexagons in A and B, respectively.

We analyzed the networks of transcription factors (TFs) and miRNAs with sKGs to identify regulatory factors at both transcriptional and post-transcriptional levels. Initially, we selected the top-ranked three transcription factors (SMAD5, ATF1, NR2F1) as regulators of sKGs based on two topological measures — degree (≥2) and betweenness (≥55.8), respectively (Fig 3A). Next, we selected the top two miRNAs (hsa-mir-34a-5p and hsa-mir-1-3p) as post-transcriptional regulators of sKGs based on degree (≥5) and betweenness (≥348.53) as topological criteria (Fig 3B).

### 3.6. Revealing common pathogenetic processes of T2D and KC with sDEGs

We examined common functional terms and pathways of both T2D and KC by the functional enrichment analyses for 6 sKGs (Table 3). In the significantly enriched GO terms (BPs, MFs, CCs) and KEGG pathways are involving sDEGs and sKGs, which are associated the pathogenic processes of T2D in KC.

**Table 3:** GO-terms and KEGG pathways linked to T2D and KC that are noticeably enhanced.

	GO ID	GO-Terms	sDEGs (counts)	Bonferron	FDR	*P.v*alues	sKGs associated
**Biological processes** **(BPs)**	GO:0043123	“Positive regulations of canonical NF-κB signal transduction”	6	0.044	0.058	8.46E-04	CD74, TFRC
	GO:1990830	“Cellular reaction to leukemia inhibitory factor”	4	0.059	0.03	0.004	CREB1, TFRC
	GO:0043066	“Inhibition of apoptotic processes”	7	0.02	0.03	0.005	CD74, CREB1, TFRC, MCL1
	GO:0030890	“Enhancement of B cell proliferation”	3	0.06	0.05	0.009	CD74, TFRC
	GO:0010595	“Positive regulation of endothelial cell-migration”	3	0.07	0.04	0.01	SCARB1
**Molecular Function (MFs)**	GO:0005515	“Protein binding”	61	5.34E-06	5.34E-06	2.61E-08	SCARB1, TFRC, CREB1, MCL1, CD74, JUN
	GO:0003723	“RNA binding”	**13**	0.03	0.02	0.002	JUN, TFRC
	GO:0042802	“Identical protein binding”	14	0.05	0.02	0.003	CD74, JUN, TFRC, CREB1,
	GO:0042803	“Activity of protein homodimerization”	8	0.08	0.05	0.01	TFRC, MCL1
	GO:0023026	“MHC-II protein complex binding”	4	0.06	0.07	0.0084532251	CD74, TFRC
**Cellular Components (CC)**	GO:0070062	“Extracellular exosome”	24	3.15E-05	3.07E-05	1.83E-07	CD74, SCARB1, TFRC,
	GO:0005829	“Cytosol”	32	0.06	0.03	4.12E-04	MCL1,
	GO:0030666	“Endocytic vesicle membrane”	4	0.02	0.07	0.001	SCARB1, CD74
	GO:0005737	“Cytoplasm”	26	0.06	0.04	0.04	SCARB1, MCL1, CD74,
	GO:0016020	“Membrane”	25	0.05	0.03	0.01	SCARB1, TFRC, CD74, MCL1
**KEGG Pathway**	hsa04380	“Osteoclast differentiation”	4	0.05	0.06	0.01	JUN, CREB1
	hsa04621	“NOD-like receptor signaling pathway”	4	0.06	0.04	0.03	JUN
	hsa05167	“Kaposi sarcoma-associated herpesvirus infection”	4	0.08	0.06	0.03	JUN, CREB1
	hsa04668	“TNF signaling pathway”	3	0.09	0.06	0.05	JUN, CREB1
	hsa04926	“Relaxin signaling pathway”	3	0.01	0.06	0.05	JUN, CREB1

### 3.7. Methylation analysis of sKGs in KC

DNA methylation is an epigenetic mechanism that regulates gene expression [88]. It enables researchers to select potential biomarkers for early diagnosis, prognosis, and therapies [89]. DNA methylation of key genes helps researchers learn about how key cellular processes are regulated and how they can disruspt the expression of sKG, making it an important part of genomic research [90]. We used methsurv web-tool to check the DNA methylation status at CpG sites for all sKGs (JUN, CD74, TFRC, CREB1, MCL1, SCARB1). All of the sKGs had multiple significant CpG sites, as we observed (*p*-value of ≤0.01) (Table S6 in S1 File). We observed that two CpG sites (cg00543485 and cg24946133) in JUN are biologically relevant to transcriptional regulation, since in these sites JUN is hyper methylated and survival probability curves are significantly separated based on its low and high expressions. Similarly, a CpG site of CD74, SCARB1 and TFRC are biologically relevant to transcriptional regulation. The other two key-genes (MCL1 and CREB1) are significantly methylated but the status of biological relevance to transcriptional regulation are not available in the methsurv-database (Table S6 in S1 File, Figure S3 in S1 File). Upregulated genes are often associated with promoter hypomethylation, particularly in cancer. Hypomethylation in promoter regions can lead to increased gene expression, contributing to tumor development and progression [91]. On the other hand, downregulated genes are often associated with promoter hypermethylation, a common epigenetic mechanism that silences gene expression, particularly in cancer [92].

### 3.8. Homology modeling

**Fig 4:**
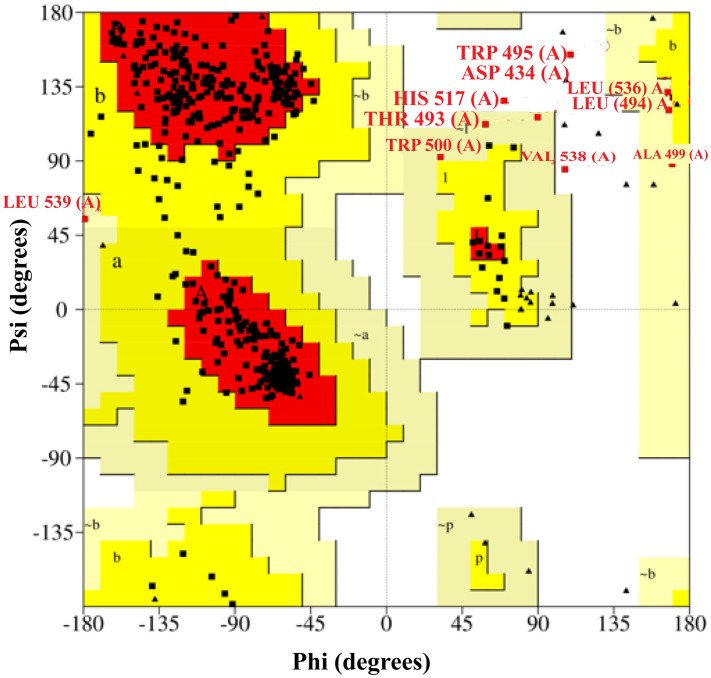
The Ramachandran plot illustrates the phi-psi angles for each residue of beta-tubulin. The red areas show the most favorable phi-psi angle combinations. The white area shows an unfavorable phi-psi combination.

SWISS-MODEL was used to generate the 3D structure of SCARB1, and the global model quality estimation (GMQE) score was 0.84. In general, a GMQE score of 0.70 or higher is considered a reliable predictor [93]. Furthermore, it was demonstrated that the SCARB1 crystal structure and the template protein exhibited 100% identity. The QMEANDisCo score for the SCARB1, calculated using SWISS-MODEL, was 0.60 ± 0.05. ProCheck performed a Ramachandran plot analysis to evaluate the stereochemical quality of the protein structure by assessing phi and psi torsion angles. In Fig 4 showed that all residues fell the preferred region (92.36%) while none fell within the inappropriate region. As a result, the model has been validated as high quality and used for further ligand–receptor interaction studies.

### 3.9. Molecular docking

**Fig 5:**
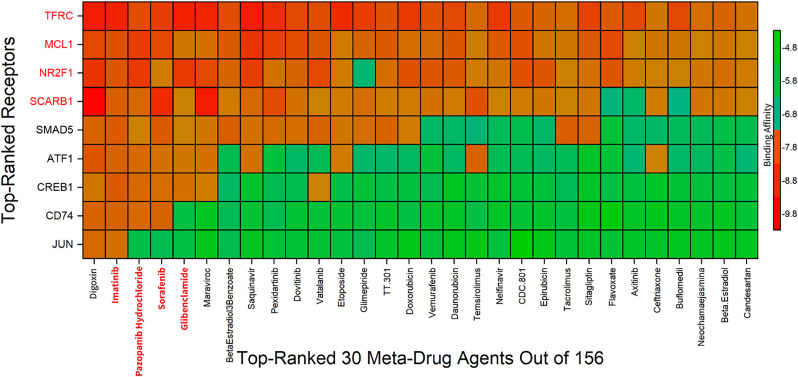
The molecular docking score matrix displays strong binding affinities between target proteins and drug agents represented in red, while weak bindings are shown in green. The X-axis represents the top 30 ranked drug agents (selected from 156), while the Y-axis shows the proposed receptors in order.

For exploring the candidate drugs for T2D and KC we considered 6 sKGs (CD74, TFRC, CREB1, MCL1, SCARB1 and JUN) and 3 regulatory TFs proteins (SMAD5, ATF1, NR2F1) and 156 drug receptors which has collected from Database and published article (Table S1 in S1 File). The 3D structures of CD74, TFRC, CREB1, MCL1, SCARB1, JUN, SMAD5, ATF1, and NR2F1 were downloaded from PDB database and SWISS-MODEL with codes E7EQJ3, 60KD, 5ZK1, 3WIX, Q8WTV0, 1JUN, 6FZS, P18846 and F1DAL7. The PubChem database used for downloaded the 3D structures of drug receptors. Then we applied molecular docking simulation between our 9 proposed protein and 156 drug agents. The binding affinity score matrix between the top ordered proposed protein receptors with top ordered 30 drug-ligands were presented in Fig 5 and Table S7 in S1 File. To look at the effectiveness of the top ranked five drugs in terms of resistance against the most up-to-date alternative receptors for T2D and KC. The binding affinity scores of top five drugs (Digoxin, Imatinib, Pazopanib hydrochloride and Sorafenib and Glibenclamide) average were −9.2, −8.1, −8.6, −8.9 and −9.1 (kcal/mol) respectively. The top ordered five drugs for proposed protein with 3D view, protein and ligand interaction and amino acid residues are shown in (Table S8 in S1 File). These drugs may be promising candidates for treating T2D and KC than the currently available drugs.

### 3.10. Re-docking of co-crystalized ligand with RMSD

**Fig 6:**
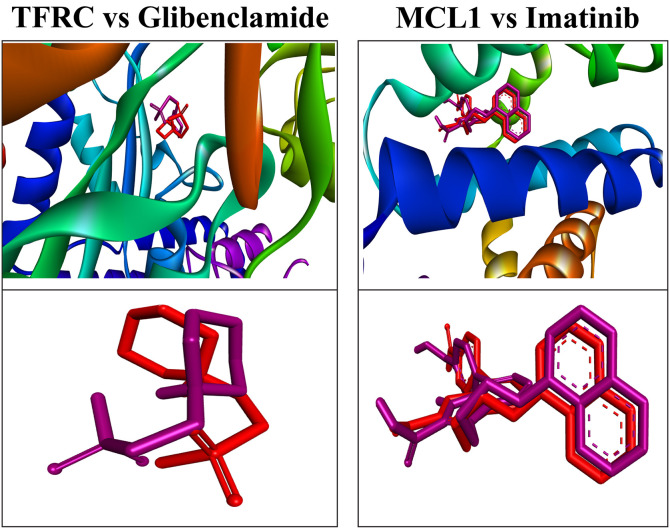
To confirm the accuracy of the docking procedure, the co-crystallized ligand structure from the TFRC, and MCL1 (PDB ID: 60KD, and 3WIX) was re-docked. As demonstrated, the docked ligand (red) closely resembles the crystallized ligand (purple) (RMSD = 1.398 Å, and 0.539 respectively).

To validate the docking study, the co-crystal ligand in the binding site of the PDB file is typically re-docked, and the RMSD of the docking poses is calculated relative to the co-crystal pose. This comparison assesses the alignment between the experimental ligand position and the docked poses. The co-crystal ligand must be re-docked at the binding site, followed by calculating the RMSD to compare it with the original co-crystal pose. In this study, the RMSD of the co-crystal ligand was calculated (Fig 6) to compare two different poses, revealing that none of the molecules had an RMSD exceeding the acceptable range, with all values remaining under 2.0 Å. The RMSD values obtained by re-docking the co-crystal ligands were 1.398 Å for Imatinib, and 0.539 Å for Glibenclamide.

### 3.11. Drug likeness and ADME/T analysis

Table 4 shows that our recommended top four drug compounds (Glibenclamide, Imatinib, Pazopanib hydrochloride, and Sorafenib) satisfy nearly all of the drug-likeness criterion. To assess the suggested drugs’ efficacy and indemnity, different parameters can be used to analyze their toxicity and ADME analyses. The compounds are anticipated to have adequate absorption in the gastrointestinal tract, suggesting they could be promising candidates for oral drugs. A substance is regarded as highly absorbed in the human gut if its Human Intestinal Absorption (HIA) score is high that is HIA ≥ 30% [94,95]. Our suggested drug molecules all had strong HIA scores of at least ≥ 65%, indicating that the body could effectively absorb them.

**Table 4:** Drug-likeness profiles of the top-ranked five drug molecules.

Drug molecules	Molecular weight (MW)	Log-P	Hydrogen-bond Acceptor (HBA)	Hydrogen-bond donor (HBD)	Polar-Surface area (Å2)	Number of rotatable bonds
Digoxin	780.949	2.218	14	6	323.322	7
Pazopanib hydrochloride	474.99	3.56	8	2	192.297	5
Imatinib	493.615	4.590	7	2	216.956	7
Sorafenib	464.83	5.54	4	3	185.111	5
Glibenclamide	494.13	3.67	5	3	198.647	8

**Fig 7:**
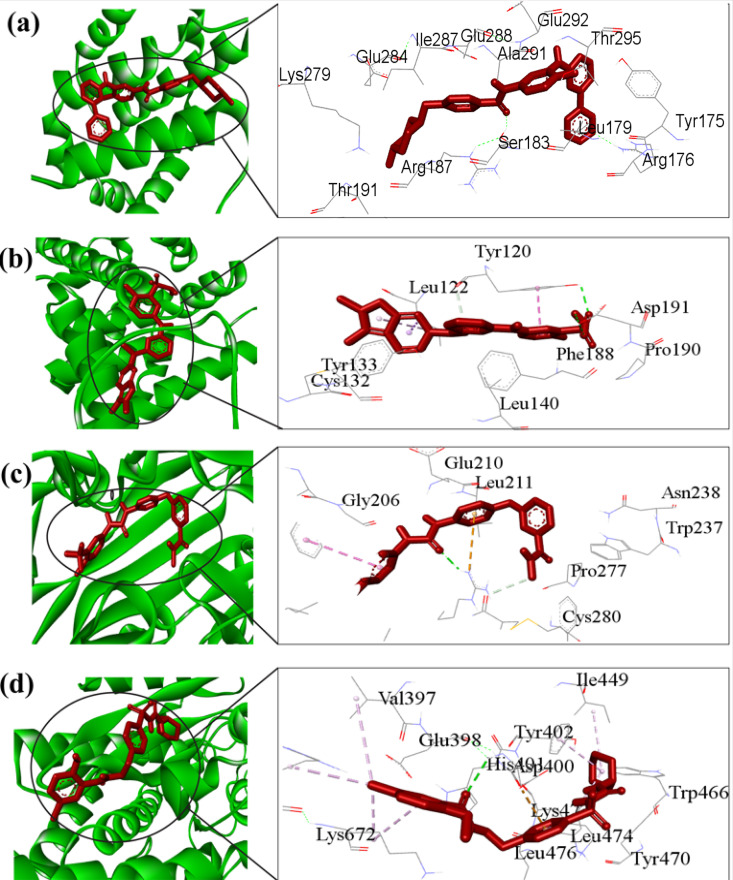
Some important docking results with the protein-ligand complexes. Top-ranked four drug-target complexes highlighting their 3-dimension (3D) view and interacting residues. Complexes: (a) indicated MCL1-Imatinib, **(b)** NR2F1- Pazopanib hydrochloride, **(c)** SCRB1-Sorafenib, and **(d)** TFRC- Glibenclamide.

A compound’s blood-brain barrier (BBB) permeability index indicates its capacity to traverse the BBB. Compounds with a LogBB value of less than <−1 are thought to be poorly disseminated through to the BB barrier, whilst compounds with a value of more than 0.3 may be able to cross the BBB with easy. Following analysis, it was determined that each drug compound had a low BBB passage ability (Table 5). The toxicity of drug molecules decreases with increasing LC50 value; conversely, decreasing LC50 value denotes increasing toxicity. Our proposed compounds’ toxicity assessments (AMES, lethal dosage LD50, and Minnow toxicity (LC50)) showed that they were inactive for all of the toxicity prediction criteria used, and as a result, they were exhibiting low toxicity based on in silico AMES and LD50 analyses, these predictions require further validation through in vitro and in vivo assays. Since digoxin does not follow the Lipinski rules, we have excluded digoxin from the suggested drug list. Thus, we observed that top-ranked four drug molecules (Glibenclamide, Imatinib, Pazopanib hydrochloride, and Sorafenib) comply most of the pharmacokinetic properties and Lipinski rules. Therefore, we considered these four drug molecules for further investigation. The 3D View of four drug molecules and their complex are display in Fig 7 and Table S8 in in S1 File. Consequently, the three drugs that have been suggested may be effective inhibitors of the genes that cause T2D and KC.

**Table 5:** ADME and Toxicity (ADME/T) profile of the top-ranked five drug molecules.

Drug molecules	Absorption (A)			Distribution (D)		Metabolism(M)	Excretion (E)	Toxicity (T)		
	Caco2 Permeability	HIA (%)	P-gpI	BBB (LogBB)	CNS LogPS	CYP3A4 Inhibitor	TC	AMES	LC_50_ (log mM)	LD_50_ (mole/kg)
				(Permeability)						
**Digoxin**	0.596	68.50	Yes	−1.387	−3.819	No	0.479	No	4.343	3.62
**Pazopanib hydrochloride**	0.344	92.23	Yes	−1.326	−2.475	Yes	−0.453	No	2.364	2.68
**Imatinib**	1.082	93.84	Yes	−1.376	−2.514	Yes	0.716	No	2.89	2.9
**Sorafenib**	0.762	85.49	Yes	−1.473	−2.025	Yes	−0.21	No	−0.515	2.14
**Glibenclamide**	0.709	71.77	Yes	−1.01	−2.674	Yes	−0.155	No	0.173	1.71

## 4. Discussion

**Fig 8:**
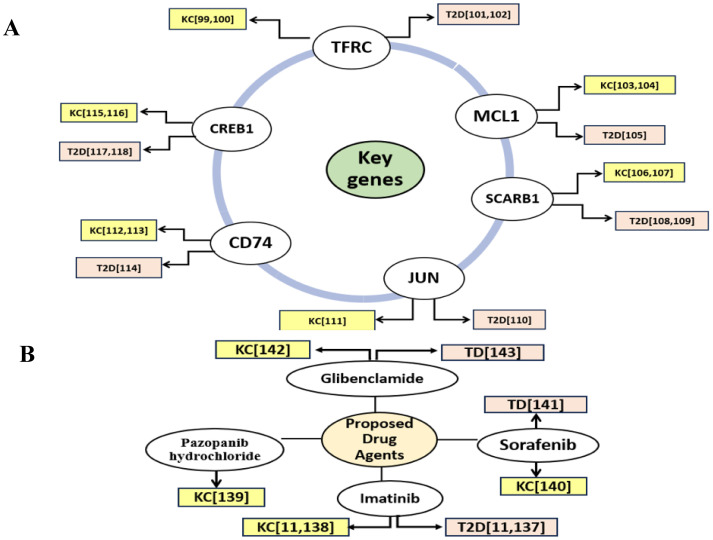
Verification of the suggested shared key genes (sKGs) and potential therapeutic agents for T2D and KC through the literature review. **(A)** Verification of the suggested T2D and KC-causing sKGs **(B)** Verification of the suggested drug-agents.

Type 2 diabetes (T2D) is a long-term metabolic disorder marked by a gradual deterioration in β-cell function over time [96]. It is considered as one of the most type of risk factor for kidney cancer (KC) [19,97,98]. Therefore, it is crucial to identify the shared key genes (sKGs) that cause both diseases in order to study their shared pathogenetic mechanisms (sPM) and potential medications for improved diagnosis and treatment when they are co-occurrence. Nonetheless, no studies have explored the pathogenic mechanisms of sKGs or potential drug candidates as a unified treatment approach for T2D and KC. Although every disease is unique, multiple drugs may also create toxicity or unfavorable side effects for patients due to drug-drug interactions [27–30]. This study investigates the genetic relationship between T2D and KC by identifying shared differentially expressed genes (sDEGs) that distinguish these conditions from control samples, with the goal of exploring common drugs as potential treatments for both T2D and KC. We investigated into 74 shared DEGs (sDEGs) in order to find common candidate genes for T2D and KC utilizing an integrated bioinformatics-based approach. The link between sKGs, T2D, and KC is further supported by several individual studies, including TFRC [99–102], MCL1 [103–105], SCARB1 [106–109] JUN [110,111] CD74 [112–114],CREB1 [115–118], as shown in Fig 8A.

The transferrin receptor (TFRC) gene encodes a transmembrane glycoprotein that functions as a cell surface receptor essential for cellular iron uptake. Through a process called receptor-mediated endocytosis, the receptor moves iron from the outside to the interior of the cell, which is crucial for cell growth. In the human renal mesangial cells (HRMCs), TFRC was overexpressed. TFRC facilitates the endocytosis of iron ions from ferritin in the blood, improving absorption [99,100]. Conversely, Patients with T2D have a considerably have greater expressed TFRC gene [101,102]. TFRC gene is regulated by three transcriptional regulators (ATF1, NR2F1 and SMAD5) and two post-transcriptional regulators (has-mir-34a-5p and hsa-mir-1-3p) of sKGs. ATF1 is a transcription factor that is a member of the ATF/CREB family. It binds to the “TGACGTCA” consensus ATF/CRE site. ATF1 might be a novel therapeutic target for the treatment of KC [119]. NR2F1’s role in controlling the expression of genes involved in cell cycle regulation, metastasis, and angiogenesis, making it an important target for understanding RCC’s molecular mechanisms and potential therapeutic interventions [120]. NR2F2, play significant roles in metabolic processes and cellular functions related to diabetes [121]. In KC, the dysregulation of miR-34a-5p has been linked to its ability to modulate oncogenes and tumor suppressor genes. It can inhibit cancer progression by targeting key pathways involved in cell survival and proliferation [122]. A study demonstrated that downregulation of miR-34a-5p improved lipid metabolism and reduced liver fat accumulation in a mouse model of T2D [123]. The gene, myeloid cell leukemia-I (MCL-1) gene also significant role in primary stages of cancer. The Bcl-2 family contains the anti-apoptotic protein Mcl-1, which is highly expressed in numerous human cancer cell lines and up-regulated in a range of human cancer, including KC [103,104]. The degradation of Mcl-1 may indeed be a decisive step in the process leading to β-cell apoptosis in both Type 1 Diabetes (T1D) and T2D [105]. Genes related to cholesterol metabolism, such as SCARB1, are significant role in the accurate diagnosis and treatment of KC [106,107]. There are many functions for SCARB1, especially in T2D [108,109]. The sKGs, CD74 expression in clear cell renal cell carcinoma (ccRCC) cells has shown to be significantly associated with high infiltration of CD4 + T cells, which may have important implications for the tumor microenvironment and immune response [112,113]. CD74 expression is upregulated in the kidneys of individuals with T2D, especially in podocytes and tubular epithelial cells. High glucose levels and pro-inflammatory cytokines like TNF-α enhance CD74 expression in these cells. When activated by macrophage migration inhibitory factor (MIF), CD74 initiates signaling pathways involving ERK1/2 and p38 MAPK, leading to increased production of inflammatory mediators such as MCP-1 and TRAIL. This inflammatory cascade contributes to the development and progression of diabetic nephropathy, a common complication of T2D [113,124]. The oncogene JUN (part of the AP-1 transcription factor) links to T2D by promoting inflammation and cellular stress pathways that impair insulin signaling and damage pancreatic beta cells. This contributes to insulin resistance and reduced insulin production, which are key in T2D development [125,126]. JUN as a key regulatory hub in RCC using a network pharmacology approach. The study suggests that gut microbial metabolites target JUN via the IL-17 signaling pathway, influencing RCC progression [127]. CREB1 promotes carcinogenesis through a major impact on tumor cell growth, proliferation, survival, metastasis, and invasion. The RCC tissues and cell lines exhibited overexpression of CREB1 [115,116]. The cAMP activating transcription factor family’s cyclic adenosine monophosphate (cAMP) response element binding protein 1 (CREB1) is essential to the regulation of gluconeogenic processes, lipid metabolism, and insulin signaling pathways. For the pathophysiology and progression of T2D, CREB1 may be a promising causal gene [117,118].

We identified the top 5 GO-terms (each of BPs, CCs and MFs) and top 5 KEGG-pathways for investigation of sKGs pathogenesis process. The top BPs such as, positive regulation of canonical NF- κB signal transduction, the negative regulation of apoptotic process, the positive regulation of B cell proliferation etc. of T2D and KC was backed up by some number of additional investigations. Among the BPs, Targeting the NF-κB pathway has become an area of interest for potential therapeutic approaches in RCC, with the hope that inhibiting this pathway could reduce tumor progression and improve patient outcomes [128]. On the other hand, NF-κB plays a central role in the pathogenesis of diabetes and its associated complications, particularly through its involvement in chronic inflammation, which is a key feature of both T1D and T2D [129]. The top CCs such as, extracellular exosome, Endocytic vesicle membrane, cytosol etc. was supported by others investigation. Among the CCs, Exosomes, small extracellular vesicles secreted by various cell types, including cancer cells, have emerged as important players in the diagnosis and treatment of RCC [130]. Exosomes derived from individuals with T2D play a significant role in mediating detrimental effects, particularly by promoting inflammation, insulin resistance, and contributing to diabetic complications [131]. NOD-like receptors (NLRs) signaling pathway, Osteoclast differentiation, Kaposi sarcoma-associated herpesvirus infection, TNF signaling pathway, Relaxin signaling pathway were top 5 KEGG pathways was also supported by others articles. Among the KEGG pathways, NLRs are a class of intracellular sensors that detect metabolic stress, pathogens, and other danger signals. They play an significant role in the body’s immune response, especially in metabolic diseases such as obesity, insulin resistance, and T2D [132]. NLRs are found in various kidney tissues during pathological conditions, indicating their important role in a range of kidney diseases, including acute kidney injury (AKI), diabetic nephropathy (DN), obstructive nephropathy (ON), lupus nephritis (LN), IgA nephropathy, uric acid nephropathy, crystal nephropathy and RCC [133]. The NLRs pathway and NF-κB signaling are central to inflammation and are implicated in both metabolic diseases and cancer. Chronic activation of NF-κB contributes to insulin resistance in T2D and promotes tumorigenesis by enhancing cell survival and proliferation. Similarly, aberrant NLRs signaling can drive inflammation that supports cancer development [134,135]. The methylation of DNA is an epigenetic process where the methyl group is added to cytosine bases, particularly at the CpG sites. DNA methylation analysis revealed that most of the sKGs were hypomethylated, leading to their increased activity as oncogenes [136]. In our investigation, we found that upregulated (CD74, TFRC, CREB1, MCL1, SCARB1 and JUN) were particularly (*p-*value of <0.01) hypomethylated in different CpG locations (Table S6 in S1 File).

Molecular docking study was utilized to investigate sKGs-guided common therapeutic compounds for both T2D and KC. The top four drug molecules (Imatinib [12,137,138], Pazopanib hydrochloride [139], Sorafenib [140,141] and Glibenclamide [142,143]) demonstrated strong binding affinities with the sKGs-mediated target proteins. These molecules were then verified for both T2D and KC through a literature review, as shown in Fig 8B. We performed ADME/T analysis and assessed drug-likeness to validate the drug-molecules computationally. Most of the identified drug molecules satisfied Lipinski’s rule of five, demonstrating their drug-like properties (Table 4). The four drug molecules that were chosen for analysis showed favorable ADME/T profiles (Table 5), high HIA percentages ranging from 68.58% to 93.85%, sufficient water solubility, and no carcinogenic characteristics. The literature review provided further support for the potential effectiveness of our suggested drugs as treatments for T2D, and KC individually. All our proposed drugs were supported as common candidate molecules for T2D and KC by individual studies on these conditions. According to the research article, imatinib successfully lowers blood sugar levels and treats T2D in the animal model [137]. Imatinib has been shown in another experimental investigation to be a highly effective inhibitor of advancer renal carcinoma [138]. Glibenclamide is a second-generation sulfonylurea that lowers blood sugar by stimulating insulin release from pancreatic beta cells. It is a well-established drug used in the treatment of T2D [142]. In a rat model of chronic kidney disease (CKD) induced by an adenine-rich diet, glibenclamide administration improved kidney structure and function. The study observed reductions in blood urea nitrogen (BUN), plasma creatinine, and proteinuria levels. Additionally, glibenclamide decreased markers of inflammation and oxidative stress in kidney tissues [143]. Pazopanib hydrochloride is a multitargeted tyrosine kinase inhibitor (TKI) that was approved by the U.S. Food and Drug Administration (FDA) for the therapy or treatment of patients with advanced RCC [139]. Sorafenib was indeed the first oral antiangiogenic multikinase inhibitor approved for the treatment of advanced RCC [140]. Sorafenib targets pathways involved in tumor growth and angiogenesis, but its interaction with metabolic conditions like T2D may alter its efficacy and toxicity [141]. Therefore, our suggested four candidate’s drugs Imatinib, Pazopanib hydrochloride, Sorafenib and Glibenclamide may be helpful for T2D and KC treatment.

## 5. Conclusion

This research combinedly investigated four transcriptomics datasets both KC (GSE15641, GSE38424) and T2D (GSE29226, GSE25724) and suggested six shared significant genomic biomarkers (JUN, CD74, TFRC, CREB1, MCL1, SCARB1) by Limma approach, pathways, as well as regulators for prognosis, diagnosis and therapies of KC patients with T2D. The shared key gene (sKGs) set enrichment analysis revealed that sKGs are highly related to some KEGG pathways (e.g., NOD-like receptor signaling pathway), as well as GO-terms BPs (e.g., Positive regulation of canonical NF-κB signal transduction) CCs (Extracellular exosome) and MFs (MHC class II protein complex binding) that are connected with T2D and KC. sKGs vs microRNAs co-regulatory vs transcription factor (TFs) investigation discovered three transcriptional regulator (SMAD5, ATF1, NR2F1) and two post transcriptional regulators(hsa-mir-34a-5p and hsa-mir-1-3p) of sKGs. DNA methylation analysis indicated that, sKGs were found to be significant at various CpG sites. Four top-ranked drug molecules (Pazopanib hydrochloride, Imatinib, Sorafenib and Glibenclamide) were suggested through molecular docking analysis with sKGs. ADMET analysis results satisfied the pharmacokinetics properties of the proposed drug-molecules. Thus, the results of this study could be significant in developing an effective common treatment strategy for both KC and T2D.

## 6. Limitation of the study

Independent transcriptomics datasets on KC and T2D were analyzed in this study to identify both diseases causing sKGs through which T2D stimulate KC, since necessary datasets were not available in the online databases that needs to be generated from the patients during the co-occurrence/existence of both diseases. However, these in-silico results might be further Improved through single cell RNA-Seq (scRNA-Seq) profile and multi-OMICS data analysis. A key limitation in this study is that the proposed sKGs between T2D and KC as well as the proposed therapeutic agents as the common treatment for both diseases, were not yet validated experimentally. Therefore, further experimental validation is necessary to confirm the results of this study. However, the results of this study might be useful input for wet-lab researchers to find effective diagnostic and therapeutic biomarkers by reducing time and cost, to adopt an appropriate treatment strategy for KC patients with T2D as comorbidity.

## Supporting information

S1 File Supplementary File Plos One. (DOCX)
