# Supplementary material for: Common molecular links and therapeutic insights between type 2 diabetes and kidney cancer
Source: PLoS One. 2025 Aug 20;20(8):e0330619. doi: 10.1371/journal.pone.0330619 (PMC12367126; doi:10.1371/journal.pone.0330619)
Supplement: S1 File — (DOCX) [file pone.0330619.s001.docx]

**Supplementary File**

**Common Molecular Links and Therapeutic Insights between Type 2 Diabetes and Kidney Cancer**

Reaz Ahmmed^1^, Mohammad Amirul Islam^2^, Md Taohid Hasan^2^, Arnob Sarker^1,2^_,_ [Md. Ahad Ali](https://www.nature.com/articles/s41598-024-69302-w#auth-Md__Ahad-Ali-Aff1-Aff3)^1^, Md. Saiful Islam^1^, Mst. Zafrin Sultana^2^**_,_** Md. Nurul Haque Mollah^1*^

^1^Bioinformatics Lab (Dry), Department of Statistics, University of Rajshahi, Rajshahi-6205, Bangladesh.

^2^Department of Biochemistry & Molecular Biology, University of Rajshahi, Rajshahi-6205, Bangladesh.

***Corresponding Author:** E-mail: [maislam14@ru.ac.bd](mailto:maislam14@ru.ac.bd), Department of Biochemistry and Molecular Biology, University of Rajshahi, Rajshahi-6205, Bangladesh.

| **Supporting Items/Captions** | **Pages** |
| --- | --- |
| **Supplementary Figures** |  |
| **Figure S1.** The Overview of this study | **1-2** |
| **Figure S2.** (A) Expression patterns of sKGs with Boxplots by GTEx and TCGA database (B) Boxplots with Independent T2D Data | **2-3** |
| **Figure S3.** MethSurv: A web tool to perform multivariable survival analysis using DNA methylation data | **3-4** |
| **Supplementary Table** |  |
| **Table S1.** Collection of T2D and KC related candidate drugs from published articles and different online web-tools. | **4-6** |
| **Table S2.** List of upregulated and downregulated DEGs between KC and control samples based four microarray gene expression datasets (GSE15641, GSE38424) | **7-8** |
| **Table S3.** List of upregulated and downregulated DEGs between T2D and control samples based four microarray gene expression datasets (GSE29226, GSE25724) | **8-9** |
| **Table S4.** Common Genes are associated with T2D and KC by statistical LIMMA approach | **9** |
| **Table S5.** List of shared key genes (sKGs) from PPI network based on different topological measures | **10** |
| **Table S6.** The significant prognostic value of CpG in sKGs | **10** |
| **Table S7.** Docking scores (binding affinities, kcal/mol) between the proposed receptors and top ordered 30 candidate drugs (out of 156) | **10-11** |
| **Table S8.** Some important docking results with the protein-ligand complexes. | **11-12** |
| R Codes and Docking Codes parameter | **12-15** |

**Supplementary Figure**

**The workflow from transcriptomics analysis to drug discovery**

Unraveling Shared Molecular Mechanism between T2D and KC, and Therapeutic Indication: Insights from Bioinformatics and System Biology Analysis workflow is displayed in **Figure S1**.


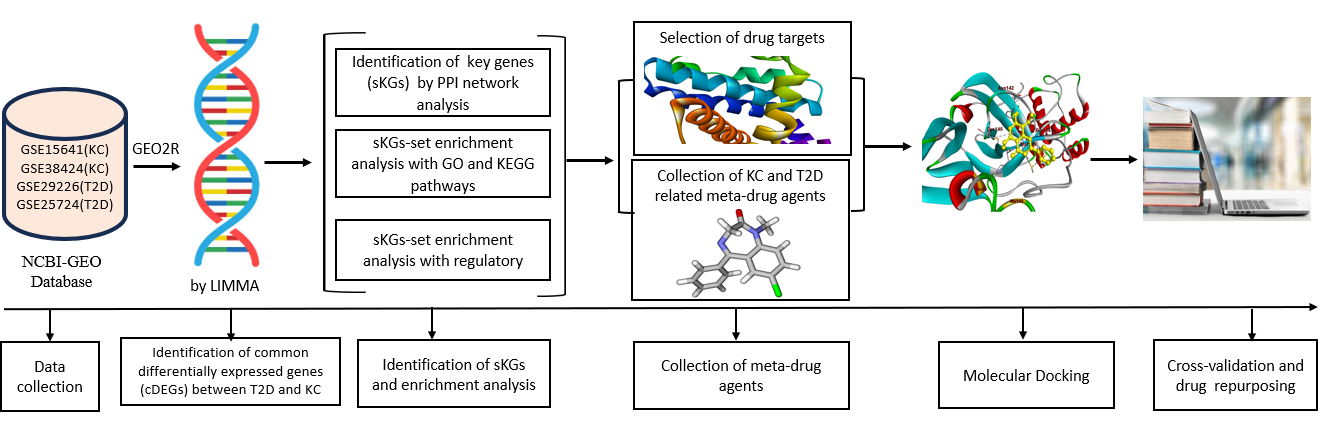


**Figure S1.** The Overview of this study

**Verification of sKGs with T2D and KC by using independent datasets and databases:**

We used box plot analysis of GTEx and TCGA data to assess sKG expression, showing that TFRC, MCL1, SCARB1, CD74, and JUN are upregulated and CREB1 is downregulated in KC (Figure S2A). Independent dataset analysis (Figure S2B) confirmed similar patterns in T2D, supporting our findings.


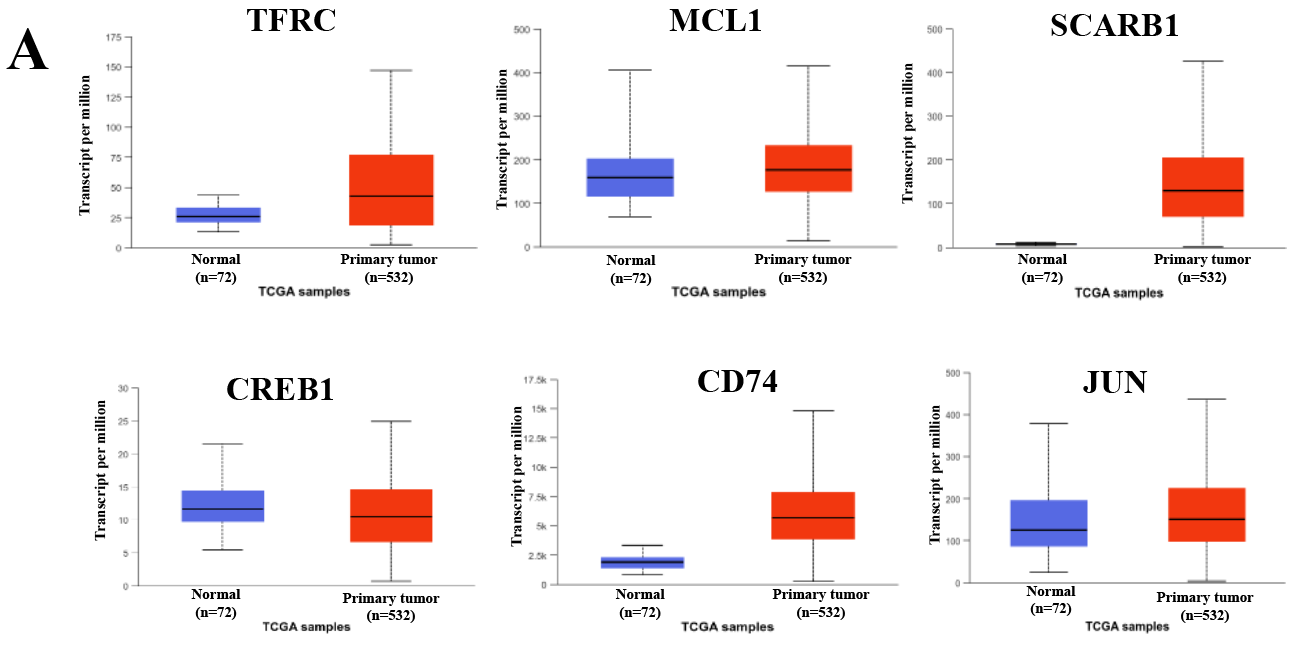


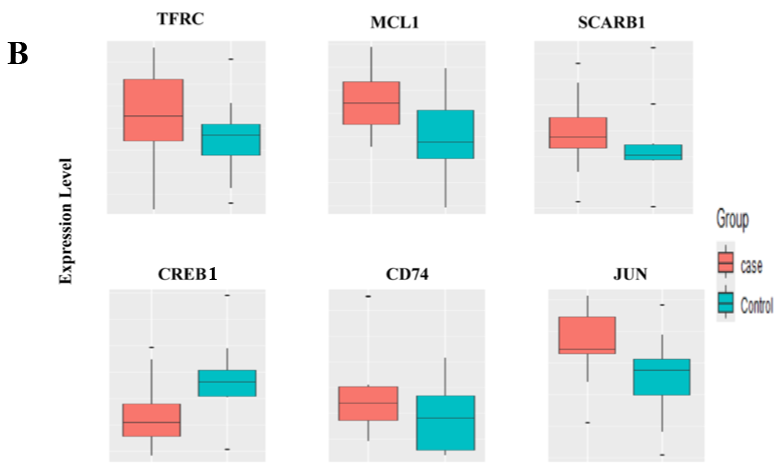


**Figure S2.** (A) Expression patterns of sKGs with Boxplots by GTEx and TCGA database (B) Boxplots with Independent T2D Data

**Methylation analysis of sKGs in KC**

**We observed that two CpG sites (**cg00543485 **and cg24946133) in JUN are biologically relevant to transcriptional regulation, since in these sites JUN is hyper methylated and survival probability curves are significantly separated based on its low and high expressions. Similarly, a CpG site of CD74, SCARB1 and TFRC are biologically relevant to transcriptional regulation. The other two key-genes (MCL1 and CREB1) are significantly methylated but the status of biological relevance to transcriptional regulation are not available in the** methsurv-database **(Table S6, Figure S3).**


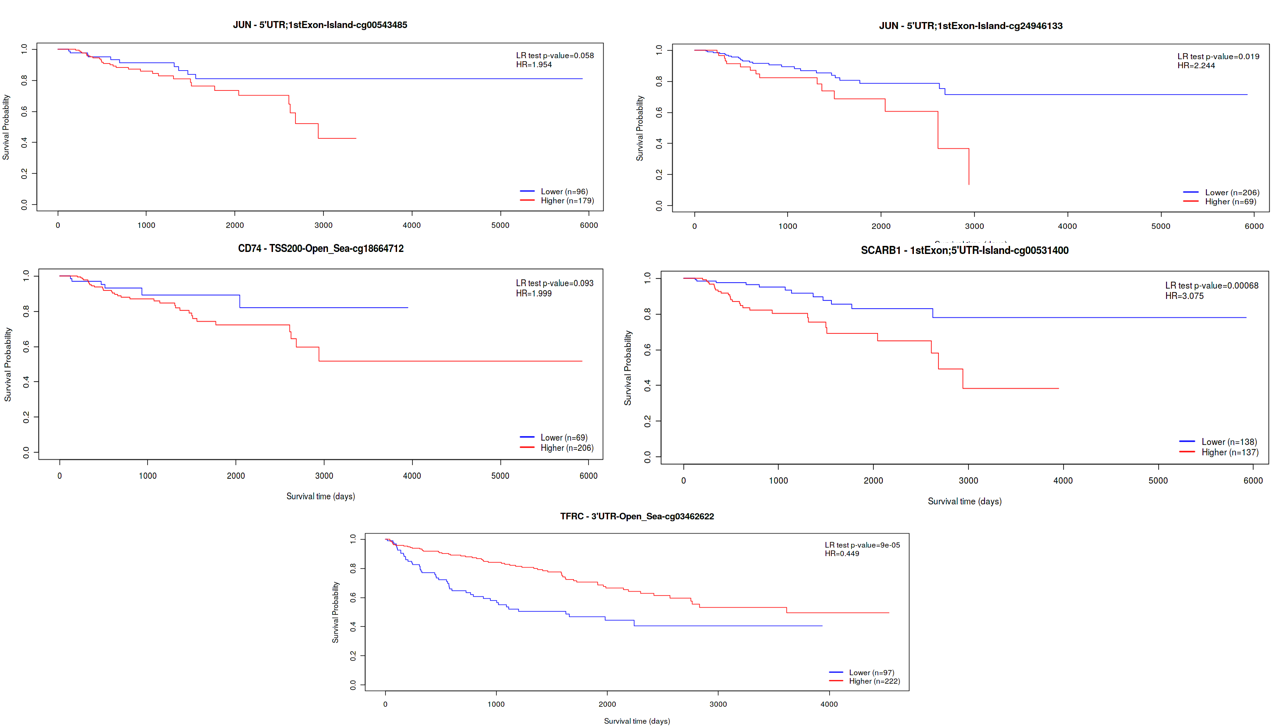


**Figure S3.** MethSurv: A web tool to perform multivariable survival analysis using DNA methylation data

**Supplementary Tables.**

**Table S1. Collection of T2D and KC related candidate drugs from published articles and** **different online web-tools.**

| **Disease type** | **Paper title with reference** | **Drug list** |
| --- | --- | --- |
| T2D | Association of pharmaceutical teachers of India a study on prescribing pattern and potential drug-drug interactions in type 2 diabetes mellitus inpatients [1] | Biguanides, Metformin, Glimepiride, Voglibose |
| T2D | Drug interactions of medications commonly used in diabetes[2] | Sulfonylureas, Meglitanides, Exenatide, Thiazolidinediones (TZDs) |
| T2D | Low utilisation of diabetes medicines in Iran, despite their affordability (2000-2012): A time-series and benchmarking study[3] | Glibenclamide, Gliclazide, Pioglitazone, Repaglinide, Acarbose, Chlorpropamide, Sitagliptin, Pioglitazone |
| T2D | Structure Based Drug Designing for Diabetes Mellitus[4] | Acetohexamide, Phenformin, Miglitol, Tolazamide, Voglibose |
| T2D | Multiple therapeutic effect of endothelial progenitor cell regulated by drugs in diabetes and diabetes related disorder[5] | Vildagliptin, Amlodipine, Aliskiren, Simvastatin |
| sKGs guided | Integration of the Drug-Gene Interaction Database (DGIdb 4.0) with open crowdsource efforts[6] | Canakinumab, Rilonacept, Tt-301, Tiludronic Acid, Risedronic Acid, Pentamidine, Thyroglobulin, Mafosfamide, Lansoprazole  , Rabeprazole, Ocriplasmin, Bruceantin, Sergeolide, Irisolidone, Neochamaejasmin A, Chembl477052, Holacanthone, Sangivamycin, Retinylretinoate, Ciprofibrate  Ranibizumab, Pegaptanib Sodium, Bevasiranib, Elmiron, Aflibercept, Bevacizumab, Cdc-801, Sildenafil, Cilostazol, Sunitinib, Resveratrol Hexanoic Acid, Eritoran, Nelfinavir, Saquinavir, Infliximab, Pravastatin, Methotrexate, Tacrolimus, Foscarnet, Cidofovir, Pamidronic Acid , Hydroquinone, Canertinib, Ionomycin, Methimazole, Leflunomide, Naproxen, Ceftriaxone, Lansoprazole, Sunitinib, Clarithromycin, Maraviroc, Leronlimab, Vicriviroc, Pexidartinib, Vatalanib, Sunitinib, Imatinib, Sorafenib,Vemurafenib, Dovitinib, Alteplase, Erythromycin, Pravastatin, Pentoxifylline, Verapamil, Cefaclor, Nicardipine, Omeprazole, Hydroquinone, Infliximab |
| sKGs guided | δ-Aminolevulinic acid-induced fluorescence unmasks biological intratumoral heterogeneity within histologically homogeneous areas of malignant gliomas[7] | Clobetasol Propionate, Mercuric Chloride, Ofloxacin, Meloxicam, Emetine, Sulfaphenazole, 6-Mercaptopurine, Amitriptyline, Beta-Estradiol 3-Benzoate, Busulfan, Thioguanine, Beta-Estradiol, Clofibrate, Chlorambucil, Digoxin, Indomethacin, Atorvastatin, Bisphenol A, Chloroxylenol, Doxorubicin, Rosiglitazone, Oxybutynin, Pralidoxime Chloride, Zidovudine, Mesna, Mebendazole, Nifedipine, Gemfibrozil, Phenacemide, Ampiroxicam, Fluphenazine, Flavoxate, Candesartan, 4-Nonylphenol, Etoposide, Streptozotocin, Myrtecaine, Venlafaxine, NN-Dimethylformamide, Norethindrone, Propylene Glycol, Benzethonium Chloride, Crotamiton, Daunorubicin, Doxifluridine, Aspirin, Buflomedil, Clonazepam, Etoposide, Trichloroethylene, Carmustine, Bithionol, Dactinomycin, Harringtonine, Nitrendipine, Praziquantel, Tetracycline, Tocainide, Azathioprine, Bis(2-Ethylhexyl)Phthalate, Epirubicin, Rofecoxib, Clonidine, 1-Naphthyl Isothiocyanate, 2-Acetylaminofluorene, Carboplatin, Clomiphene, Chlordiazepoxide, Oxyquinoline, Dexchlorpheniramine, Mitomycin, Ciprofloxacin, Captopril, Cefotaxime, Fenoprofen, Fluvastatin, Sulfadoxine |
| KC | Mechanisms of Acquired Resistance to Tyrosine Kinase Inhibitors in Clear - Cell Renal Cell Carcinoma (ccRCC) [8] | Sunitinib, pazopanib, tivozanib, sorafenib, axitinib |
| KC | As a library, NLM provides access to scientific literature. Inclusion in an NLM database does not imply endorsement of, or agreement with, the contents by NLM or the National Institutes of Health [9] | Pazopanib, Cabozantinib, Everolimus, Temsirolimus, Nivolumab |
| KC | Current and emerging therapies for first-line treatment of metastatic clear cell renal cell carcinoma [10] | Nivolumab, atezolizumab, Bevacizumab, pilimumab |
| KC | Metastatic non–clear cell renal cell carcinoma treated with targeted therapy agents: Characterization of survival outcome and application of the International mRCC Database Consortium criteria[11] | Everolimus, Temsirolimus, Tivozanib, Pazopanib, Bevacizumab, Axitinib |

**Table S2. List of upregulated and downregulated DEGs between KC and control samples based four microarray gene expression datasets (**GSE15641, GSE38424**)**

| **Downregulated cDEGs** | **Upregulated cDEGs** |
| --- | --- |
| "HADH,,KCNJ1,ACPP,GMDS,CLOCK,SMIM8,ZNF232,ALAD,ZNF839,MTHFD1,CASP2,ALDH4A1,MAP7,ALDH6A1,PCK2,RNASEL,TMEM70,FAM168A,ARSB,TDRD3,TAF15,SIRT5,SORD,PEPD,NDUFA2,SLC8A1,DCXR,MME,GIMAP5,ZNF804A,BPHL,NUDC,UBQLN4,MSRA,FBP1,CYP27B1,ALDH3A2,PTPRO,SLC7A8,LINC00339,HSDL2,VPS41,SNCA,MT1E,ZMYM3,MT1F,FECH,XRCC6,PC,TST,MRPS12,NCLN,GTF2H3,MT1G,DYRK2,PDE3B,AKR7A3,WLS,TTC38,FLI1,HSD17B14,NIN,PTPRA,GGH,ARL15,DICER1,PNPO,C14orf93,MT1H,APOO,VPS45,HS2ST1,HIBCH,MPO,GNAS,CACYBP,ACACB,RIDA,NMT1,PCCA,PROSER1,PEX1,MCM4,MT1X,RRM1,GIT2,NDST1,CEP152,SUCLG1,ECHS1,PIK3R4,ACADM,SH3YL1,C2CD2L,ADK,RFX3,PEBP1,AAK1,TACSTD2,TAB1,EMC3,MRPL19,Mar02,GPX3,ECHDC3,PKP4,R3HDM1,MAN1C1,CTSH,RAB11FIP3,KNTC1,CRYL1,RAB29,SMARCC1,DOLPP1,SLC47A1,TIMP3,USP5,POLA2,ATP5S,UCHL1,APAF1,CEP104,PIGC,SIGLEC7,TEF,CYB5A,CA2,EHBP1"  N=130 | RAD51,ZNF350,IBA57,LRRC8B,TFPI,GDF11,M6PR,TRA2A,TSC22D2,NAB2,C15orf39,SLC7A11,RPS15A,LGALS3,TRMT61A,RPL14,GCLC,RPL35A,FEM1B,MARCKS,EIF1,RPL39,CREB1,HLAE,KLF6,KIF5B,CST7,TAF13,SLC38A1,RNASE2,HLAF,RNPS1,RPL9,CSGALNACT2,HLAG,RPL31,INPP1,CPNE1,TRAF6,TMEM159,RPL24,USP7,MAGT1,PEX5,NR4A3,S100A9,UBA52,ST8SIA4,WDR1,TES,VAPA,RPLP1,MFSD5,HEY1,CTRC,CD4,COL4A3BP,ARL4A,PPP4C,RPL7,ADAM17,CELF2,RPL23A,IL36RN,CCDC59,PDCD10,PDGFA,EIF3H,CDCA4,CDKN1A,MET,CXorf40B,GFPT1,ZFR,PFKP,EEF1D,BTF3,ELF4,YTHDF3,RECQL,KDM5B,PADI4,IST1,ZNF207,RAB35,CLK3,CHMP1A,CSTA,RPS29,RNASET2,CASP4,SP2,PABPC3,TPD52L2,U2AF1,HSPA13,MAP1LC3B,ASNS,ADRM1,CYB5R3,CHIC2,PITPNA,HNRNPK,BRD2,PSD4,CD2BP2,MPZL1,BAZ1A,TWF1,USP8,RNF13,CSNK1A1,CRK,CASP3,IDS,TP53,ZPR1,DUSP10,AHR,PPP2R2A,MED28,CSNK1D,SERINC3,CD59,IL4R,SMURF1,EIF3B,STX4,GARS,ANXA2,PSMD11,PTRF,OGFR,HIST3H2A,NSMAF,USP4,MRGBP,VCP,SNX16,CAMSAP2,C5AR2,RPS27,EWSR1,ARL4C,CHMP2A,RYK,ATG101,UBE2D2,ZNF12,RBM39,SNHG17,CENPJ,DCLRE1C,SIAH1,GNAQ,SF1,LAMA5,SDS,DENR,HK1,CELF1,SNW1,ATF4,ZNF24,MAX,TNFAIP6,UBN1,PABPC1,OSER1,CD44,GTPBP4,SELL,RRN3,TRIB3,BRD9,FXYD5,EIF1AX,SAP30BP,UXT,STRN,HGF,TIMP1,RAP1B,TANK,CASP1,ARMCX6,CST3,ZNF562,TROVE2,SEC24B,RRAGC,EIF4E,C6orf48,FLOT1,SP100,JAK1,LST1,UPP1,PICALM,MAD1L1,GDI1,EPS8,CKAP4,VRK2,ISG20,AKAP13,CDC42,ITGB1,STK10,PANX1,TOR4A,RTN4,HCP5,BTG1,UBE2E3,RBPJ,ARPC5L,KMT5B,ENTPD4,UBE2Z,PDE4B,RGS1,LPCAT1,PSMD12,CAV1,RBM7,ING3,CHMP2B,SNAPC1,CHD4,CHD2,RIF1,ADA,DYNC1H1,TLR2,ISG15,HNRNPAB,SAR1A,PPP1R12A,TFIP11,ELF1,TDG,ARHGDIA,TAGLN2,AGFG1,NIPBL,HGS,EMP3,RNF219,MAP4K5,GBP2,SFPQ,MMP10,UBR4,TARS,DGKD,WIPF1,AOC4P,CXCR4,UTP3,RAN,APBA3,TNIP2,MAZ,NAMPT,HNRNPDL,C12orf10,ZCCHC6,AVL9,PLAUR,MCUB,HNRNPU,DDIT4,MAP4K4,MDM2,FAM49A,TAF7,TMEM140,CSDE1,CD53,DNAJC1,FOSL2,RBBP6,SPATA5L1,MS4A6A,CKS2,NEU1,PLAGL1,FUS,TRMO,PMAIP1,ETF1,RALGDS,SEC22B,VKORC1,EIF4G1,CTNNB1,ITGA5,SON,FBXO11,SPTLC1,SERTAD2,EIF3A,VPS4B,RYBP,SNRPB2,SP3,RARRES3,ATG12,GUK1,CTBP2,EREG,ADNP,SLC38A7,SLC1A4,CRIP1,MAP2K1,FERMT2,EZR,COPS2,PTTG1,PPARG,RB1CC1,DPM1,ECE1,CYTH1,HNRNPM,CYLD,MED13L,NKG7,TICAM1,S100A10,TNFAIP3,FAM57A,MYLIP,ANXA1,ITGAV,PEA15,Mar07,HLADPB1,C3AR1,ARL6IP1,AZIN1,ZNF395,RAE1,PPP2R3A,MALT1,ZBTB43,CLEC7A,TAF1D,BRD4,SRP19,ZYX,BZW1,GAB2,BCL2A1,NAA50,TM2D3,HLX,NRIP1,EIF6,HLADRB6,TMEM2,BIRC2,B4GALT5,NCOR2,RNF14,GNLY,DUSP4,CBLB,ICAM3,SYN2,ILF3,INSIG1,HLADPA1,RBM15,FGFR1,CAMKK2,E2F3,TUBB6,POM121C,GNAI3,SLC25A6,PLEC,TFE3,SUB1,PKIA,GLIPR1,RUNX3,SPG21,HOMER3,IRS2,SKIL,MCL1,RHEB,DNAJB1,TRIM9,MBP,SNUPN,RRAGA,SMNDC1,VEGFA,ADM,PPP1R15A,GBP1,HSPA1L,ZFX,RIOK3,IRF1,CCL5,SLC25A37,HLADRA,PLEK,PLSCR1,TESC,DUSP2,PPIF,NFAT5,CD74,Sep10,EDNRB,PLAC8,NR3C1,IRF9,PAPD7,TXNIP,NAB1,NOTCH2NL,TRIO,HECA,ADGRB1,SLC43A3,CHSY1,BLZF1,SCG2,BNIP3L,PRKCI,S100A4,PLEKHB2,DUSP14,ICAM2,ITGA2,TCEB3,ID2,CHST2,SEC14L1,IRF7,THBD,PHLDA2,SMPDL3A,SAMSN1,GRB10,RGS10,ZHX2,CCL20,TIPARP,FHL2,IFITM3,DYRK3,TNFRSF12A,DEGS1,HIC2,CITED2,HMOX1,DDX3X,LAMA2,TPM4,NDRG1,IFITM2,NFKBIA,S100P,CD9,CFD,BAALC,TNIP1,ZMYM2,EHD1,RRAD,BIRC3,SRGN  N=559 |

**Table S3. List of upregulated and downregulated DEGs between T2D and control samples based four microarray gene expression datasets (**GSE29226, GSE25724**)**

| **Downregulated cDEGs** | **Upregulated cDEGs** |
| --- | --- |
| UPF3A,USP1,SEC62,PPM1A,MKRN2,GTF3C3,G3BP2,RNF14,EXOC1,ARPP19,TPD52,AKAP11,,PFN2,UBE2E3,EID1,ASF1A,ENPP2,TTC19,ST13,KLHL18,PRKAG1,UBL3,HNRNPDL,TFB2M,OSTM1,CFAP20,ASPH,WNK1,ZNF148,RAB2A,EIF2B1,VLDLR,C6orf120,RDX,TEX2,LZTFL1,ERGIC2,CPE,ABCE1,RRAGD,MBD4,PSD3,NEK7,PLP2,USP46,LIPT1,TSR1,PRKAA1,THAP12,ZNF706,YEATS4,DERL2,TNPO1,ANKMY2,ORC3,RPL14,C12orf29,SS18L1,PAM,Sep10,SLC39A6,MAP4K3,TERF1,CREB3L2,PSMD1,SQLE,SUPT16H,DNM1L,KPNA3,BLCAP,KIF5C,ENDOD1,API5,MAN1A2,ADNP,ADO,TRIM13,KIF1BP,RUFY3,PARP2,EIF5A,MRPL49,MTR,HLTF,TOMM70,MOAP1,RHOBTB3,SLC25A36,EEF1E1,UGP2,ZMYND11,NBN,DYRK2,ITGB1BP1,TROVE2,PLEKHB2,SLC35A3,KIAA0196,POLR2C,ERP44,KCTD9,ERLIN1,YTHDC2,KAT6B,PTPRN2,SMAP1,UBE3B,MTRR,ASXL2,ABHD10,SNRPD3,ARMCX5,SDHC,CBFB,RC3H2,CREB1,CTSC,PAXBP1,SCML1,ZNF302,SPATA5L1,MKL2,SHQ1,ATP5S,TBCE,NEK3,PAAF1,FASTKD3,UBE3C,DCUN1D4,KTN1,RAD23B,PHACTR2,LRBA,WASF3,MARK1,ETFDH,GPS2,TSPYL4,RECQL,EML1,SENP6,MAP4K5,NECTIN3,C17orf75,CYLD,PRMT3,PKD2,FDX1,EPS15,ID3,ID1,YY1,PDS5B,SAP18,LEMD3,PIBF1,TSPAN12,GALNT12,CCDC91,TULP4,PRC1,SCPEP1,VPS13C,ARMC1,RAB27A,RPS6KB1,PDGFRL,APP,DENND1B,ZNF318,PAQR3,NEDD4L,NMNAT2,CHMP2B,EIF2AK2,RINT1,LIMCH1,KIAA0368,SLC35A2,CD9,PCIF1,NBEA,TMEM106B,RHOQ,ALCAM,GOLGA1,CHCHD2,GGPS1,ACO2,RAB29,SRI,METTL5,BSG,PPM1H,COBLL1,KCTD12,GOLGA8A,WLS,ZNF415,USP14,MDC1,RNF170,PDE4DIP,WSB1,Mar05,NPAT,DSG2,NSL1,PNO1,IFT57,ANKRA2,TOR3A,ZNF529,PLEKHA1,RSU1,AGAP1,ZNF83,C21orf59,RTN4,PDCD2,TMEM109,ENTPD4,MEIS2  **N=225** | HLADRB6,LST1,MYH3,DHX34,INPP5D,SS18,LAIR2,ADAM19,CACFD1,LBP,LRRN2,LAT2,LDLR,PRPH,FGFR1OP,MCL1,ATXN2L,MYH11,LRRC3,HCAR3,SOCS3,GK,ACAP1,KIAA0226L,JUN,BTG2,SLC19A1,EGR1,MMP25,PLAUR,KCNMB1,ABCA7  **N=33** |

| **Table S4. Common Genes are associated with T2D and KC by statistical LIMMA approach** |
| --- |
| SRI,RPL14,SCARB1,PKM,CREB1,TUBB,INPP5D,EIF2B1,RECQL,TCF3,RHOQ,DYRK2,IFNAR2,ANKMY2,TROVE2,PLEKHA1,METTL5,LST1,UGP2,RTN4,UBE2E3,TMEM109,ENTPD4,CHMP2B,TOR3A,ARMC1,MAP4K5,KCTD12,PARP12,NAMPT,HNRNPDL,PDCD2,PLAUR,SPATA5L1,SON,RTN3,CCDC91,ADNP,CD52,CYLD,HMGN2,AZIN1,QPCT,TM2D3,HLADRB6,RSU1,RNF14,INSIG1,MCL1,TFRC,VEGFA,SLC39A6,CD74,Sep10,NSL1,PLEKHB2,ZNF428,CD9,BSG,BST2,RUSC2,ALDH4A1,RNASEL,WLS,CACYBP,ZNF669,AAK1,RAB29,PNO1,TIMP3,ATP5S,ARID5B,JUN=74 |

**Table S5. List of shared key genes (sKGs) from PPI network based on different topological measures**

| **SN** | **List of shared key genes (sKGs) from PPI network based on different topological measures** | | | | | | | |
| --- | --- | --- | --- | --- | --- | --- | --- | --- |
| 1 | **SKGs** | **Betweenness** | **BottleNeck** | **Closeness** | **EPC** | **MNC** | **Radiality** | **Stress** |
| 2 | JUN | 996.9303 | 16 | 52.5 | 34.69 | 36 | 4.6029 | 3326 |
| 3 | CD74 | 413.4999 | 6 | 45.83333 | 33.804 | 25 | 4.39705 | 1802 |
| 4 | CREB1 | 596.0716 | 11 | 47.83333 | 34.275 | 28 | 4.45588 | 2140 |
| 5 | MCL1 | 318.665 | 9 | 42.16667 | 32.413 | 18 | 4.27941 | 530 |
| 6 | SCARB1 | 497.265 | 7 | 38.56 | 33.175 | 21 | 4.26470 | 554 |
| 7 | TFRC | 324.76 | 7 | 43.58333 | 32.898 | 21 | 4.27941 | 1266 |

| **Table S6. The significant prognostic value of CpG in sKGs** | | | | | |  |  |
| --- | --- | --- | --- | --- | --- | --- | --- |
| **sKGs** | **Gene Group** | **CpG Island** | **Name of CPG sites** | **HR** | **CPG site *P*-values** | **Survival *p-*value** | **Methylation status** |
| **JUN** | **5'UTR;1stExon** | **Island** | **cg00543485** | **0.419** | **0.000213** | **0.058** | **Hyper** |
| JUN | 3'UTR;1stExon | Island | cg09414137 | 0.42 | 0.001569 | NA | NA |
| JUN | 1stExon | Island | cg12271199 | 0.333 | 4.18E-07 | NA | NA |
| JUN | TSS1500 | Island | cg15995771 | 0.466 | 0.000214 | NA | NA |
| JUN | TSS1500 | S_Shore | cg20787340 | 0.499 | 0.001078 | NA | NA |
| **JUN** | **5'UTR;1stExon** | **Island** | **cg24946133** | **0.297** | **5.20E-05** | **0.019** | **Hyper** |
| CD74 | Body | Open_Sea | cg13362637 | 4.048 | 2.88E-05 | NA | NA |
| **CD74** | **TSS200** | **Open_Sea** | **cg18664712** | **0.389** | **0.001037** | **0.00025** | **Hyper** |
| CD74 | TSS1500 | Open_Sea | cg19966212 | 2.643 | 0.001562 | NA | NA |
| CREB1 | 5'UTR | Island | cg05727155 | 0.426 | 5.19E-05 | NA | NA |
| CREB1 | 5'UTR;1stExon | Island | cg06691214 | 0.411 | 0.001471493 | NA | NA |
| CREB1 | 5'UTR | Island | cg14237317 | 0.42 | 0.00198734 | NA | NA |
| MCL1 | 1stExon | Island | cg09804220 | 0.41 | 0.000858617 | NA | NA |
| MCL1 | Body | N_Shore | cg24998145 | 2.057 | 0.000342629 | NA | NA |
| **SCARB1** | **1stExon;5'UTR** | **Island** | **cg00531400** | **0.396** | **6.63E-05** | **2.4e-06** | **Hyper** |
| SCARB1 | Body | S_Shore | cg05620762 | 0.465 | 0.000189925 | NA | NA |
| SCARB1 | Body | Open_Sea | cg10911287 | 0.476 | 0.00031439 | NA | NA |
| SCARB1 | Body | N_Shore | cg15282973 | 3.619 | 1.16E-08 | NA | NA |
| SCARB1 | TSS200 | Island | cg15283062 | 0.467 | 0.000674326 | NA | NA |
| SCARB1 | Body | S_Shelf | cg17514098 | 0.452 | 6.47E-05 | NA | NA |
| SCARB1 | Body | Open_Sea | cg22775642 | 3.584 | 0.000128578 | NA | NA |
| SCARB1 | Body | Open_Sea | cg23460943 | 3.471 | 0.000361106 | NA | NA |
| **TFRC** | **3'UTR** | **Open_Sea** | **cg03462622** | **0.488** | **0.000244545** | **9E-05** | **Hyper** |
| TFRC | 5'UTR;1stExon | Island | cg14641705 | 0.373 | 5.33E-05 | NA | NA |
| TFRC | Body | Open_Sea | cg23502966 | 0.472 | 0.000220559 | NA | NA |
| TFRC | 1stExon;5'UTR | Island | cg27335386 | 0.425 | 0.001724165 | NA | NA |

[Note: NA indicate Not Available]

| **Table S7:** **Docking scores (binding affinities, kcal/mol) between the proposed receptors and top ordered 30 candidate drugs (out of 156)** | | | | | | | | | |
| --- | --- | --- | --- | --- | --- | --- | --- | --- | --- |
|  | TFRC | MCL1 | NR2F1 | SCARB1 | SMAD5 | ATF1 | CREB1 | CD74 | JUN |
| Digoxin | -9.2 | -8.3 | -8.7 | -9.8 | -7.7 | -8 | -7.3 | -7.7 | -7.6 |
| Imatinib | -9.1 | -8.1 | -8 | -7.8 | -7.9 | -7.7 | -7.9 | -7.7 | -7.4 |
| PazopanibHydrochloride | -8.2 | -8.5 | -8.6 | -7.6 | -7.1 | -7.6 | -7.6 | -7.6 | -6 |
| Sorafenib | -8.5 | -8.3 | -7.2 | -8.9 | -7.9 | -7.4 | -7.5 | -7.7 | -6.1 |
| Glibenclamide | -9.1 | -7.3 | -8.6 | -7.1 | -7.1 | -7.3 | -7.4 | -5.9 | -5.9 |
| Maraviroc | -9 | -7.5 | -8.3 | -9.2 | -7.3 | -7.4 | -7.2 | -5.3 | -5.2 |
| BetaEstradiol3Benzoate | -8.2 | -7.9 | -7.7 | -7.2 | -7.8 | -6.1 | -6.4 | -6.2 | -6.1 |
| Saquinavir | -9.2 | -8.7 | -8.5 | -7.6 | -7.4 | -7.4 | -5.5 | -5.6 | -5.3 |
| Pexidartinib | -8.8 | -8.4 | -7.4 | -8.2 | -7.6 | -5.8 | -6.1 | -6.1 | -5.6 |
| Dovitinib | -8.3 | -8.2 | -7.7 | -7.4 | -7.5 | -6.5 | -6.1 | -5.6 | -5.7 |
| Vatalanib | -8.2 | -8 | -8.3 | -7.1 | -7.7 | -6.2 | -7.1 | -5.7 | -5.6 |
| Etoposide | -8.9 | -7.2 | -7.2 | -7.2 | -7.3 | -7.2 | -6 | -5.6 | -5.8 |
| Glimepiride | -8.5 | -7.7 | -6.7 | -7.5 | -7.5 | -6.6 | -5.7 | -5.8 | -6.1 |
| TT-301 | -8.1 | -7.9 | -7.5 | -7.2 | -7.7 | -6.6 | -5.9 | -5.5 | -5.5 |
| Doxorubicin | -8.6 | -7.5 | -8.1 | -7.2 | -7.2 | -6.4 | -5.8 | -5.9 | -5.1 |
| Vemurafenib | -8.1 | -7.6 | -8 | -7.4 | -6.5 | -5.7 | -6.1 | -5.5 | -5.7 |
| Daunorubicin | -8.3 | -8 | -7.9 | -7.2 | -6.5 | -6.4 | -5.3 | -5.4 | -5.2 |
| Temsirolimus | -7.6 | -7.2 | -7.2 | -8.1 | -6.8 | -7.7 | -5.6 | -5.9 | -5.1 |
| Nelfinavir | -8.6 | -7.9 | -7.3 | -7.1 | -6.3 | -6.2 | -5.5 | -5.4 | -5.8 |
| CDC-801 | -7.9 | -8.1 | -8.1 | -7.2 | -6.1 | -6.2 | -5.7 | -5.7 | -4.7 |
| Epirubicin | -7.7 | -7.3 | -8 | -7.3 | -6.6 | -6.4 | -5.8 | -5.5 | -5.1 |
| Tacrolimus | -7.5 | -7.3 | -7.1 | -7.1 | -7.8 | -6.2 | -6.2 | -5.8 | -5.7 |
| Sitagliptin | -8.4 | -7.9 | -7.4 | -7.3 | -7.7 | -5.4 | -5.4 | -5.1 | -5.4 |
| Flavoxate | -7.9 | -8.3 | -8 | -6.9 | -5.7 | -5.7 | -5.9 | -4.9 | -5.6 |
| Axitinib | -8.3 | -7.1 | -7.2 | -6.6 | -6.6 | -6.7 | -5.8 | -5.4 | -5.4 |
| Ceftriaxone | -7.3 | -7.3 | -7.1 | -7.2 | -6.5 | -7.1 | -5.4 | -5.2 | -5.4 |
| Buflomedil | -8.3 | -7.3 | -7.1 | -7 | -6.3 | -6.6 | -5.6 | -5.4 | -5.3 |
| Neochamaejasmina | -7.5 | -7.1 | -7.3 | -7.3 | -6.3 | -6.4 | -5.8 | -5.5 | -5.3 |
| Beta Estradiol | -7.7 | -7.3 | -7.5 | -7.3 | -6 | -6 | -5.8 | -5.3 | -5.2 |
| Candesartan | -7.4 | -7.1 | -7.2 | -7.1 | -6.1 | -6.7 | -5.7 | -5.7 | -5.3 |

| **Table S8.** Some important docking results with the protein-ligand complexes. First, second and third columns indicate target proteins, drug molecules and their binding affinities, respectively. Fourth and fifth column indicate 2D and 3D view of the drug-target complexes, and 6^th^ column indicates the interacting amino acids in the complexes. | | | | | |
| --- | --- | --- | --- | --- | --- |
| **Potential Target** | **Structure of the Compounds** | **Binding Affinity score (kCal/mol)** | **Target-Ligand Interaction (2D-view)** | **Target-Ligand Interaction (3D-view)** | **Interacting of the Amino Acids** |
| **TFRC** | 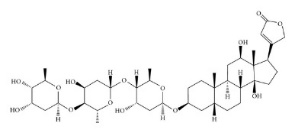  Digoxin | **-9.2** | 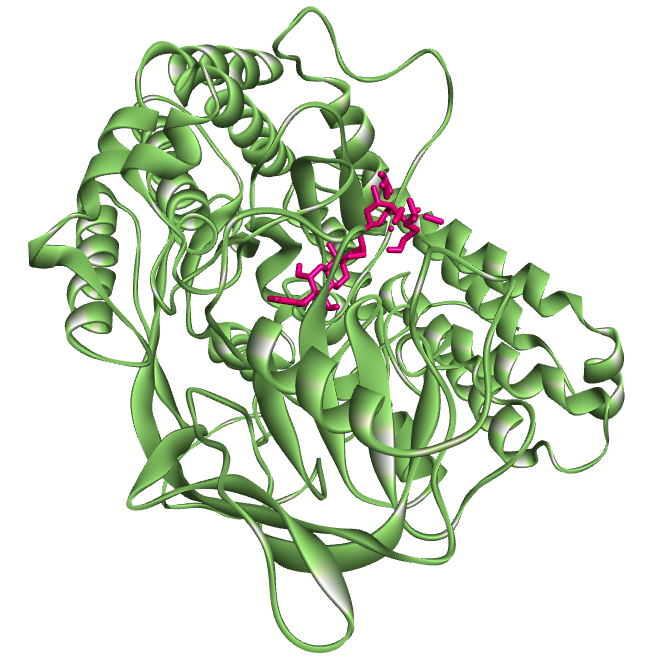 | 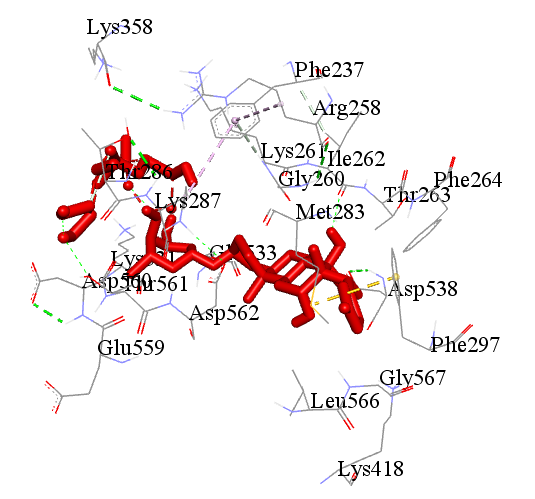 | Lys358,Phe237,Arg258, Phe264,Thr263,Asp538, Phe297,Gly567,Leu566, Lys418,Glu559,Asp560,Lys287,Glu533, Ile262,Gly260,Thr561 |
| **TFRC** | 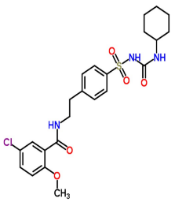Glibenclamide | **-9.1** | 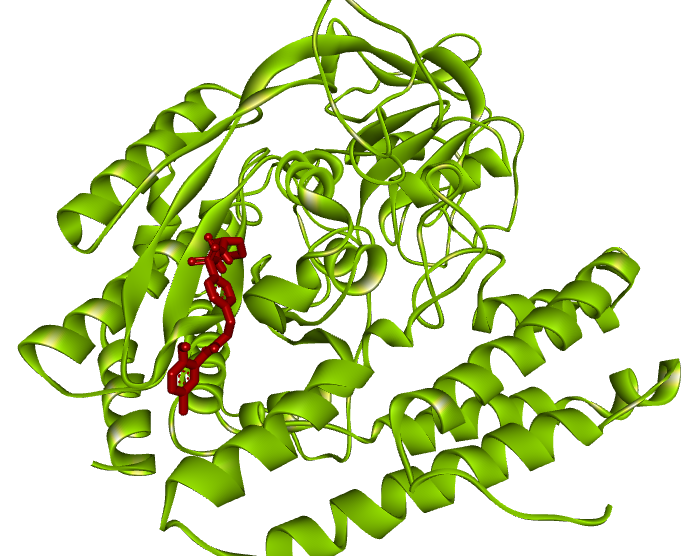 | 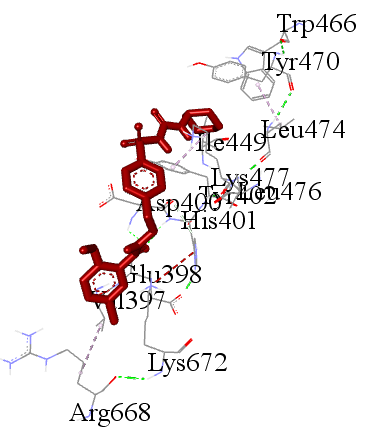 | Trp466,Tyr470,Leu474,Leu476,Lys477,Ile449,Asp400,His401,Tyr402,Glu398,Val397,Lys672,Arg668 |
| **MCL1** | 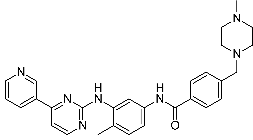    Imatinib | **-8.1** | 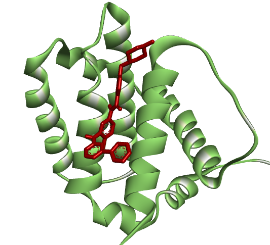 | 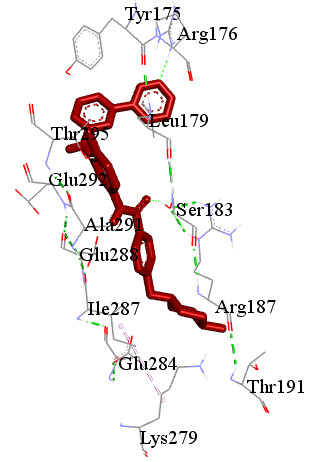 | Tyr175,Arg176,Leu179, Ser183,Arg187, Thr191, Lys279, Glu284, Ile287, Glu288,Ala291,Glu292, Thr295 |
| **NR2F1** | 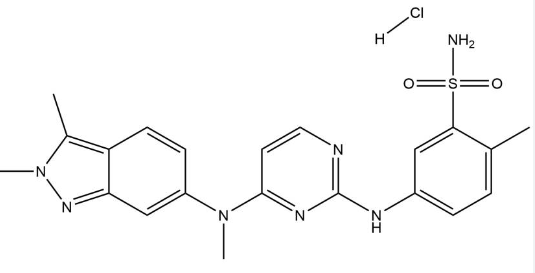  Pazopanib hydrochloride | **-8.6** | 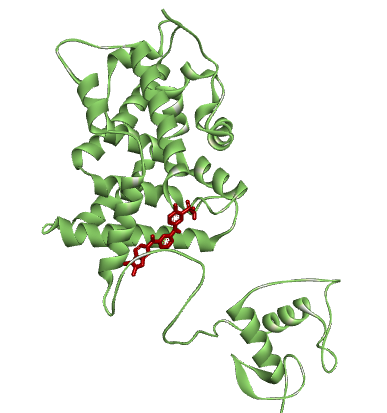 | 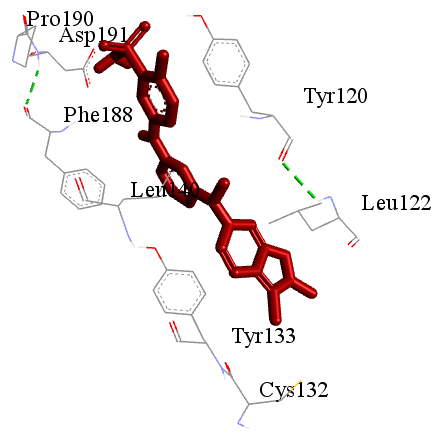 | Pro190,Asp191,Phe188,Tyr120,Leu122,Tyr166,Cys132,Leu140 |
| **SCARB1** | 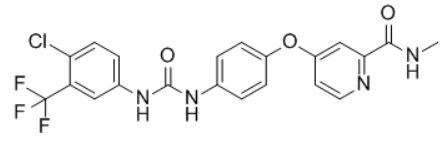  Sorafenib | **-8.9** | 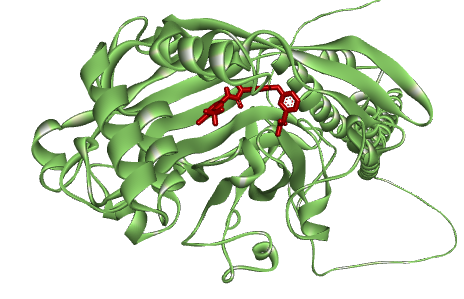 | 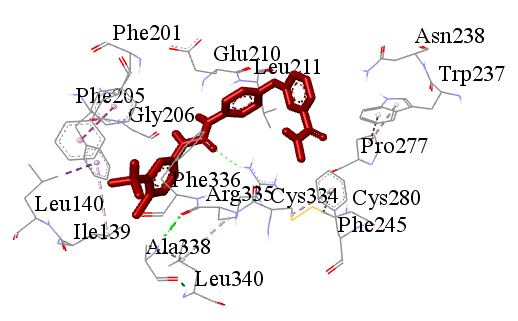 | Phe205,Gly206,Glu210,Leu211,Leu140,Ile139,Asn238,Trp237,Pro277,Cys280,Cys234,Leu340,Ala338,Arg335 |

**CODES**

1. **For sDEGs identification**

library(dplyr)

#Setup your working directory

setwd("")

# Load the dataset

results1 =read.csv("GSE15641.csv", header=TRUE)

results2 =read.csv("GSE38424.csv", header=TRUE)

results3 =read.csv("GSE25724.csv", header=TRUE)

results4 =read.csv("GSE29226.csv", header=TRUE)

##### GSE1

Upregulated_data41 <- results1 %>% filter( adj.P.Val <0.05 & logFC >1)

dim(Upregulated_data41)

#write.csv(Upregulated_data41 ,file="UPDE.csv")

##Select Downregulated genes

Downregulated_data41 <- results1 %>% filter( adj.P.Val <0.05 & logFC < -1)

dim(Downregulated_data41)

#write.csv(Downregulated_data41 ,file="DownDE_1.csv")

#### GSE2

##Select Upregulated genes

Upregulated_data38424 <- results2 %>% filter( adj.P.Val <0.05 & logFC >1)

dim(Upregulated_data38424)

#write.csv(Upregulated_data38424 ,file="UPDE.csv")

##Select Downregulated genes

Downregulated_data38424 <- results2 %>% filter( adj.P.Val <0.05 & logFC < -1)

dim(Downregulated_data38424)

#write.csv(Downregulated_data38424,file="DownDE_1.csv")

#### GSE3

##Select Upregulated genes

Upregulated_data24 <- results3 %>% filter( adj.P.Val <0.05 & logFC >1)

dim(Upregulated_data24)

#write.csv(Upregulated_data24 ,file="UPDE.csv")

##Select Downregulated genes

Downregulated_data24 <- results3 %>% filter( adj.P.Val <0.05 & logFC < -1)

dim(Downregulated_data24)

#write.csv(Downregulated_data24,file="DownDE_1.csv")

##### GSE4

Upregulated_data26 <- results4 %>% filter( adj.P.Val <0.05 & logFC >1)

dim(Upregulated_data26)

#write.csv(Upregulated_data26 ,file="UPDE.csv")

##Select Downregulated genes

Downregulated_data26 <- results4 %>% filter( adj.P.Val <0.05 & logFC < -1)

dim(Downregulated_data26)

#write.csv(Downregulated_data26 ,file="DownDE_1.csv")

UP_41 <- unique(Upregulated_data41$Gene.symbol)

UP_38424 <- unique(Upregulated_data38424$Gene.symbol)

UP_24 <- unique(Upregulated_data24$Gene.symbol)

UP_26 <- unique(Upregulated_data26$Gene.symbol)

common_DEG = Reduce(intersect, list(UP_41,UP_38424,UP_24,UP_26))

Down_41 <- unique(Downregulated_data41$Gene.symbol)

Down_38424 <- unique(Downregulated_data38424$Gene.symbol)

Down_24 <- unique(Downregulated_data24$Gene.symbol)

Down_26 <- unique(Downregulated_data26$Gene.symbol)

common_DEG = Reduce(intersect, list(Down_41,Down_38424,Down_24,Down_26))

DEG_41 = c(UP_41, Down_41)

DEG_38424= c(UP_38424, Down_38424)

DEG_24 = c(UP_24, Down_24)

DEG_26 = c(UP_26, Down_26)

common_DEG = Reduce(intersect, list(DEG_41,DEG_38424,DEG_24,DEG_26))

length(common_DEG)

write.csv(common_DEG,file="Common_gene set.csv")

1. Docking Parameter

**********************************************************

receptor = CD74_main.pdbqt

center_x = 12.678

center_y = -5.014

center_z = 18.332

size_x = 104

size_y = 108

size_z = 106

num_modes = 10

energy_range = 4

*****************************************************************************

#!/usr/bin/perl

print"Enter filename of ligands list:\t";

$ligfile=<STDIN>;

chomp $ligfile;

open (FH,$ligfile)||die "Cannot open file\n";

@arr_file=<FH>;

for($i=0;$i<@arr_file;$i++)

{print"@arr_file[$i]\n";

@name=split(/\./,@arr_file[$i]);

}for($i=0;$i<@arr_file;$i++)

{chomp @arr_file[$i];

print"@arr_file[$i]\n";

system("vina.exe --config conf.txt --ligand @arr_file[$i] --log @arr_file[$i]_log.log");

}

**References**

[1] S. Manjusha, M. Amit, S. Ronak, Association of pharmaceutical teachers of India a study on prescribing pattern and potential drug-drug interactions in type 2 diabetes mellitus inpatients, Indian J. Pharm. Pract. 7 (2014) 7–12.

[2] C. Triplitt, Drug interactions of medications commonly used in diabetes, Diabetes Spectr. 19 (2006) 202–211. https://doi.org/10.2337/diaspect.19.4.202.

[3] A. Sarayani, A. Rashidian, K. Gholami, Low utilisation of diabetes medicines in Iran, despite their affordability (2000-2012): A time-series and benchmarking study, BMJ Open. 4 (2014) 1–9. https://doi.org/10.1136/bmjopen-2014-005859.

[4] K. Ramanathan, H. Karthick, N. Arun, Structure Based Drug Designing for Diabetes Mellitus, J. Proteomics Bioinforma. 3 (2010) 310–313. https://doi.org/10.4172/jpb.1000157.

[5] R.K. Ambasta, H. Kohli, P. Kumar, Multiple therapeutic effect of endothelial progenitor cell regulated by drugs in diabetes and diabetes related disorder, J. Transl. Med. 15 (2017) 1–17. https://doi.org/10.1186/s12967-017-1280-y.

[6] S.L. Freshour, S. Kiwala, K.C. Cotto, A.C. Coffman, J.F. McMichael, J.J. Song, M. Griffith, O.L. Griffith, A.H. Wagner, Integration of the Drug-Gene Interaction Database (DGIdb 4.0) with open crowdsource efforts, Nucleic Acids Res. 49 (2021) D1144–D1151. https://doi.org/10.1093/nar/gkaa1084.

[7] A. V. Moiyadi, E. Sridhar, δ-Aminolevulinic acid-induced fluorescence unmasks biological intratumoral heterogeneity within histologically homogeneous areas of malignant gliomas, Acta Neurochir. (Wien). 157 (2015) 617–619. https://doi.org/10.1007/s00701-014-2321-4.

[8] Z. Bielecka, A. Czarnecka, W. Solarek, A. Kornakiewicz, C. Szczylik, Mechanisms of Acquired Resistance to Tyrosine Kinase Inhibitors in Clear - Cell Renal Cell Carcinoma (ccRCC), Curr. Signal Transduct. Ther. 8 (2014) 219–228. https://doi.org/10.2174/1574362409666140206223014.

[9] B. Baishya, A. Satpathy, R. Nayak, R. Mohanty, As a library , NLM provides access to scientific literature . Inclusion in an NLM database does not imply endorsement of , or agreement with , the contents by NLM or the National Institutes of Health . Learn more : PMC Disclaimer | PMC Copyright Notice, 23 (2019) 163–167.

[10] M.B. Atkins, N.M. Tannir, Current and emerging therapies for first-line treatment of metastatic clear cell renal cell carcinoma, Cancer Treat. Rev. 70 (2018) 127–137. https://doi.org/10.1016/j.ctrv.2018.07.009.

[11] N. Kroeger, W. Xie, J.L. Lee, G.A. Bjarnason, J.J. Knox, M.J. MacKenzie, L. Wood, S. Srinivas, U.N. Vaishamayan, S.Y. Rha, S.K. Pal, T. Yuasa, F. Donskov, N. Agarwal, C.K. Kollmannsberger, M.H. Tan, S.A. North, B.I. Rini, T.K. Choueiri, D.Y.C. Heng, Metastatic non-clear cell renal cell carcinoma treated with targeted therapy agents: Characterization of survival outcome and application of the International mRCC Database Consortium criteria, Cancer. 119 (2013) 2999–3006. https://doi.org/10.1002/cncr.28151.
